# Supplementary material for: Practices and trends in clinical trial registration in the Pan African Clinical Trials Registry (PACTR): a descriptive analysis of registration data
Source: BMJ Open. 2022 Jan 25;12(1):e057474. doi: 10.1136/bmjopen-2021-057474 (PMC8796231; doi:10.1136/bmjopen-2021-057474)
Supplement: Author's manuscript [file bmjopen-2021-057474.draft_revisions.pdf]

BMJ Open

BMJ Open is committed to open peer review. As part of this commitment we make the peer review history of every article we publish publicly available.

When an article is published we post the peer reviewers' comments and the authors' responses online. We also post the versions of the paper that were used during peer review. These are the versions that the peer review comments apply to.

The versions of the paper that follow are the versions that were submitted during the peer review process. They are not the versions of record or the final published versions. They should not be cited or distributed as the published version of this manuscript.

BMJ Open is an open access journal and the full, final, typeset and author-corrected version of record of the manuscript is available on our site with no access controls, subscription charges or pay-per-view fees (http://bmjopen.bmj.com).

If you have any questions on BMJ Open's open peer review process please email info.bmjopen@bmj.com

BMJ Open

A trends analysis of clinical trial registration in PACTR

Journal:	*BMJ Open*
Manuscript ID	bmjopen-2021-057474
Article Type:	Original research
Date Submitted by the Author:	16-Sep-2021
Complete List of Authors:	Ndwandwe, Duduzile; South African Medical Research Council<br>Runeyi, Sinazo; South African Medical Research Council, Cochrane South Africa<br>Pienaar, Elizabeth ; South African Medical Research Council, Cochrane South Africa<br>Mathebula, Lindi; South African Medical Research Council, Cochrane SA<br>Hohlfeld, Ameer ; South African Medical Research Council, Cohrane South Africa<br>Wiysonge, Charles; South African Medical Research Council, Cochrane South Africa
Keywords:	Clinical trials < THERAPEUTICS, PUBLIC HEALTH, Public health < INFECTIOUS DISEASES

SCHOLARONE™
Manuscripts

I, the Submitting Author has the right to grant and does grant on behalf of all authors of the Work (as defined in the below author licence), an exclusive licence and/or a non-exclusive licence for contributions from authors who are: i) UK Crown employees; ii) where BMJ has agreed a CC-BY licence shall apply, and/or iii) in accordance with the terms applicable for US Federal Government officers or employees acting as part of their official duties; on a worldwide, perpetual, irrevocable, royalty-free basis to BMJ Publishing Group Ltd ("BMJ") its licensees and where the relevant Journal is co-owned by BMJ to the co-owners of the Journal, to publish the Work in this journal and any other BMJ products and to exploit all rights, as set out in our licence.

The Submitting Author accepts and understands that any supply made under these terms is made by BMJ to the Submitting Author unless you are acting as an employee on behalf of your employer or a postgraduate student of an affiliated institution which is paying any applicable article publishing charge ("APC") for Open Access articles. Where the Submitting Author wishes to make the Work available on an Open Access basis (and intends to pay the relevant APC), the terms of reuse of such Open Access shall be governed by a Creative Commons licence – details of these licences and which Creative Commons licence will apply to this Work are set out in our licence referred to above.

Other than as permitted in any relevant BMJ Author's Self Archiving Policies, I confirm this Work has not been accepted for publication elsewhere, is not being considered for publication elsewhere and does not duplicate material already published. I confirm all authors consent to publication of this Work and authorise the granting of this licence.

For peer review only

A trends analysis of clinical trial registration in PACTR

Duduzile Ndwandwe[1*]; Sinazo Runeyi[1]; Elizabeth D Pienaar[1]; Lindi Mathebula[1]; Ameer Hohlfeld[1]; Charles Wiysonge[1,2,3]

[1]Cochrane South Africa, South African Medical Research Council, Tygerberg, South Africa

[2]Division of Epidemiology and Biostatistics, Faculty of Medicine and Health Sciences, Stellenbosch University, Tygerberg, 7505, Cape Town, South Africa.

[3]School of Public Health and Family Medicine, Faculty of Health Sciences, University of Cape Town, Observatory, 7925, Cape Town, South Africa.

*Correspondence author: Duduzile Ndwandwe, Cochrane South Africa, South African Medical Research Council, Tygerberg, South Africa. Email: Duduzile.Ndwandwe@mrc.ac.za

For peer review only - http://bmjopen.bmj.com/site/about/guidelines.xhtml

Abstract

Background

The Pan African Clinical Trials Register (PACTR) is a World Health Organization (WHO) International Clinical Trial Registry Platform (ICTRP) primary register, which caters for clinical trials conducted in Africa. The aim is to describe and report on the trends of trial records registered in PACTR.

Methods

This is a cross-sectional study of trials registered in PACTR as of 18 August 2021. We analyzed the following data fields: study intervention, disease condition, sex of the participants, sample size, ethics, funding, and availability of results. The analysis was conducted using Microsoft Excel.

Results

The number of trials registered continues to increase, almost reaching 3000 marks (n=2998). The registration status shifted towards prospectively registered trials (55%). Ethical approval has been received in 90% of the registered trials. Factorial assignment as an intervention model was in 20% of the trials registered. There were 36% completed trials, of which 3% had results available in the registry. The most dominant funding source indicated that 23% of the registered trials were self-funded, and 55% had no funding.

Discussion

Registration on PACTR continues to grow; however, our analysis shows that researchers' capacity-building is needed to understand the importance of the registry and how this information informs healthcare decisions. Funding investments for African clinical research remain essential to ensure that the trials conducted in Africa are adequately resourced.

Conclusion

For peer review only

For peer review only - http://bmjopen.bmj.com/site/about/guidelines.xhtml

Awareness-raising to promote prospective trial registration remains critical to avoid selective reporting bias, primarily when registry information can be used in systematic reviews to inform research gaps and opportunities for funders, policymakers, patients, and researchers.

Keywords: Pan African Clinical Trial Register, Clinical trial registration, Prospective trial registration

For peer review only

For peer review only - http://bmjopen.bmj.com/site/about/guidelines.xhtml

Introduction

The Pan African Clinical Trials Registry (PACTR) was established from the AIDS, Tuberculosis, and Malaria Registry based at the South African Cochrane Centre [1-3]. The registry was established in collaboration with Cochrane's Infectious Diseases Group (CIDG), based at the Liverpool School of Tropical Medicine and the World Health Organization (WHO). PACTR is the only African member of the WHO Network of Primary Registers, which transfers trial information to the WHO International Clinical Trials Registry Platform (WHO-ICTRP) every month [4, 5]. WHO-ICTRP serves as a platform aligned with the International Committee of Medical Journal Editors (ICMJE) for prospective trial registration. PACTR contributes to regional transparency and harmonisation of clinical trial research [6, 7]. A clinical trials registry is a database in which key administrative and scientific information about planned, ongoing, and completed trials are stored [6-8]. Thus, registration of all interventional trials is considered scientific, ethical, and responsible [9-11]. Accessing clinical trials information allows informing decision-making on healthcare decisions based on all available evidence [9]. Such decisions cannot be easily made if publication bias and selective reporting exist [9]. Furthermore, the Declaration of Helsinki states that "Every clinical trial must be registered in a publicly accessible database before recruitment of the first subject" [9].

Further benefit to register trials in a registry is that it allows for similar or identical trials to be known, making it possible for researchers and funding agencies to avoid unnecessary duplication [7]. Also, describing clinical trials in progress makes it easier to identify research gaps for new research to advance the knowledge gaps. Registries checking data as part of the registration process may lead to improvements in the quality of clinical trials by making it possible to identify potential problems (such as problematic randomisation methods) early in the research process

For peer review only - http://bmjopen.bmj.com/site/about/guidelines.xhtml

There has been a push from governments and international organisations, especially since 2005, to make clinical trial information more widely available and standardise registries and registering processes. The World Health Organization (WHO) has published international Standards for Clinical Trial Registries to achieve consensus on both the minimal and the optimal operating standards for trial registration [12]. To adhere to WHO practices that ensure that collected data are not duplicated and provide meaningful information, registry staff scrutinise each application and perform regular quality checks to ensure quality data is contained in the registry.

Although in the past, research on the clinical trial landscape provided key insights into the global burden of disease, and more generally, the global and regional clinical trial landscapes [4, 7, 13, 14], before PACTR, there was no regional support for longitudinal monitoring of planned and ongoing African clinical trials. PACTR is unique in recognising that African researchers face additional challenges in trial registration and seeks to provide feasible ways of overcoming these barriers [2]. PACTR has seen substantial growth in the number of trials registered from inception until recently. In this cross-sectional survey of the PACTR database, we report on the trends in the clinical trials registered.

For peer review only - http://bmjopen.bmj.com/site/about/guidelines.xhtml

MATERIALS AND METHODS

This was a descriptive analysis of clinical trials registered in the Pan African Clinical Trial Register (PACTR).

Inclusion and exclusion criteria

The data was comprised of clinical trials registered in the PACTR and accessible to the WHO ICTRP. The advanced search function of ICTRP was used to identify these trials. The searches were run on 18 August 2021. The study used the WHO definition of clinical trial: "any research study that prospectively assigns human participants or groups of humans to one or more health-related interventions to evaluate the effects on health outcomes"[12]. The trials in the ICTRP included both registered and unregistered trials, which primary registered send data file to the ICTRP monthly. Study records with no registration number were excluded.

Data management and analysis

Data were downloaded from the PACTR and exported into an excel spreadsheet by one researcher (SR) on 18 August 2021. We searched WHO-ICTRP as a second set of data for verification purposes on the same date. All records were quality-checked by a second researcher (DN). We only excluded records with no PACTR registration number. In each record of the included trials, the following data items were used for analysis: registration status, disease condition, sex of the participants in the trials; sponsor, intervention type, funding source, the age range of participants, intervention model, phase of the trial and overall status. We conducted a trend analysis of the registered trials in PACTR to understand the pattern of trial registration over the years using Microsoft Excel.

For peer review only - http://bmjopen.bmj.com/site/about/guidelines.xhtml

Results

We report on the trends for trials registered in PACTR listed in the ICTRP portal. Data was downloaded on 18 August 2021 and found a total of 4962 trial records. Of these records, we excluded 1964 that were not registered. PACTR is the WHO primary register that sends data monthly to ensure that ICTRP has a one-stop portal to access trials records. The data sent to WHO ICTRP includes both registered trials and those pending, which can be linked directly to the registry that sent the data. The final number of trial records contained in our analysis were 2998 records that had a PACTR registration number.

PACTR has grown substantially since its inception, with each year showing a steady increase in the number of trials registered. The year 2020 had the most registered trials (n=606). We anticipate that this increase will be seen even this year(**figure 1**).

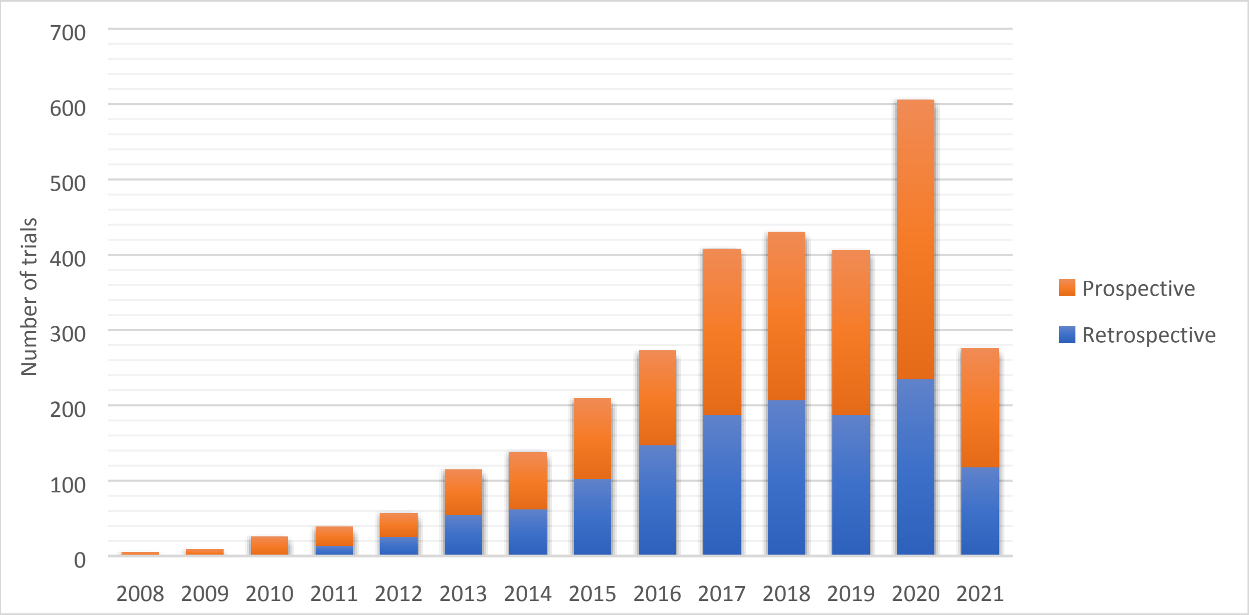

Figure 1: Number of retrospective and prospective registrations on PACTR by year

For peer review only - http://bmjopen.bmj.com/site/about/guidelines.xhtml

Table 1 shows our analysis of some of the registry data items. Generally, there has been an increase in the number of trials registered in PACTR, with n= 2998 identified at the analysis time (figure1). There are 1083 (36%) completed trials with 94 (3%) having results available in the registry. Twenty-eight percent (28%) of trials registered (836/2998) are listed as not recruiting, while 25% (755/2998) are recruiting participants. 55% (1655/2998) of the trials are registered prospectively, with the remaining 45% (1343/2998) registered retrospectively.

Our data show that most of the trials registered in PACTR have ethics approval 90% (2691/2998). The most common intervention model in the registered trials was factorial assignment 20% (589/2998). The trial phases show an almost equal distribution for all clinical trial phases. We assessed the sponsor of the registered trials and reported that the sponsor could be the funder. Our data show that 55% (1639/2998) of the trials have no funding, while 23% (693/2998) being self-funded. Many trials recruited both male and female 75% (2240/2998) participants among the registered trials. The median sample size was 1140.7, with 0 to 1087000 participants listed in the trial records.

Table 1: Characteristics of trials in PACTR

Description	*N* (%)
**Number of trials registered**	2998
**Number of studies completed**	1083 (36.1)
**Number of completed with results**	94 (3.1)
**Overall trial status**	
Completed	1083 (36.1)
Ongoing/Active	-

For peer review only - http://bmjopen.bmj.com/site/about/guidelines.xhtml

Not yet recruiting/pending	836 (27.9)
Recruiting	755 (25.2)
Stopped/terminated	-
Suspended	7 (0.2)
Withdrawn	-
Other/unknown	317 (10.6)
**Prospective/retrospective**	
Prospectively registered	1655 (55.2)
Retrospectively registered	1343 (44.8)
**Intervention model**	
Parallel assignment	2124 (70.8)
Single group assignment	59 (2.0)
Crossover assignment	201 (6.7)
Factorial assignment	589 (19.6)
Sequential assignment	13 (0.4)
None (open label)	12 (0.4)
**Phase**	
Not applicable	2310 (77.1)
Phase I	192 (6.5)
Phase II	138 (4.6)
Phase III	199 (6.6)
Phase IV	155 (5.2)
**Sponsor**	
University	196 (6.5)
Industry/NGO	61 (2.0)
Government	107 (3.6)
Charities	94 (3.1)

For peer review only - http://bmjopen.bmj.com/site/about/guidelines.xhtml

Hospital	67 (2.2)
Self-funded	693 (23.1)
Funding agency	142 (4.7)
Other	45 (1.5)
No funding	1639 (54.7)
**Ethics**	
Yes	2691 (90)
No	307 (10)
**Sex**	
Both	2240 (74.7)
Female	628 (20.9)
Male	130 (4.3)
**The target number of participants**	
Min, max	0, 1087000
Mean (SD)	1140.7 (26156.8)
Median (IQR)	80 (125)

Researchers have an option in the PACTR registry to indicate the type of intervention for the trial.

We show that most trials use drugs for treatment 21% (622/2998) as an intervention (**figure 2**).

For peer review only

For peer review only - http://bmjopen.bmj.com/site/about/guidelines.xhtml

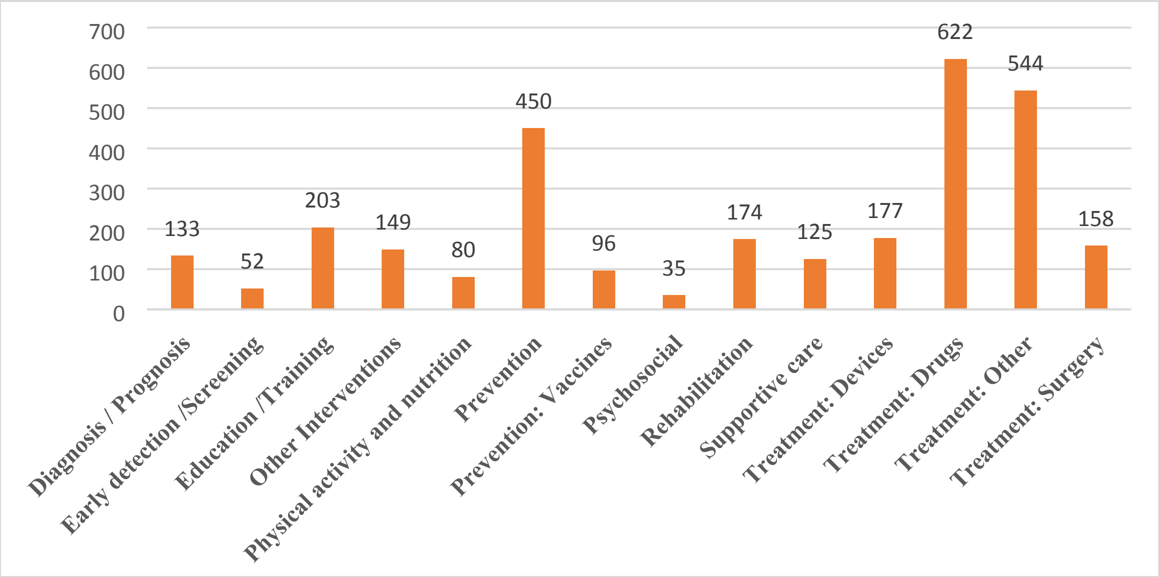

Figure 2: Type of intervention used for the registered trials in PACTR

The most common disease conditions investigated in the trials conducted in PACTR registered trials were infections and infestations with 20% (586/2998), followed by the surgery category 14% (426/2998) trials (**Figure 3**).

For peer review only - http://bmjopen.bmj.com/site/about/guidelines.xhtml

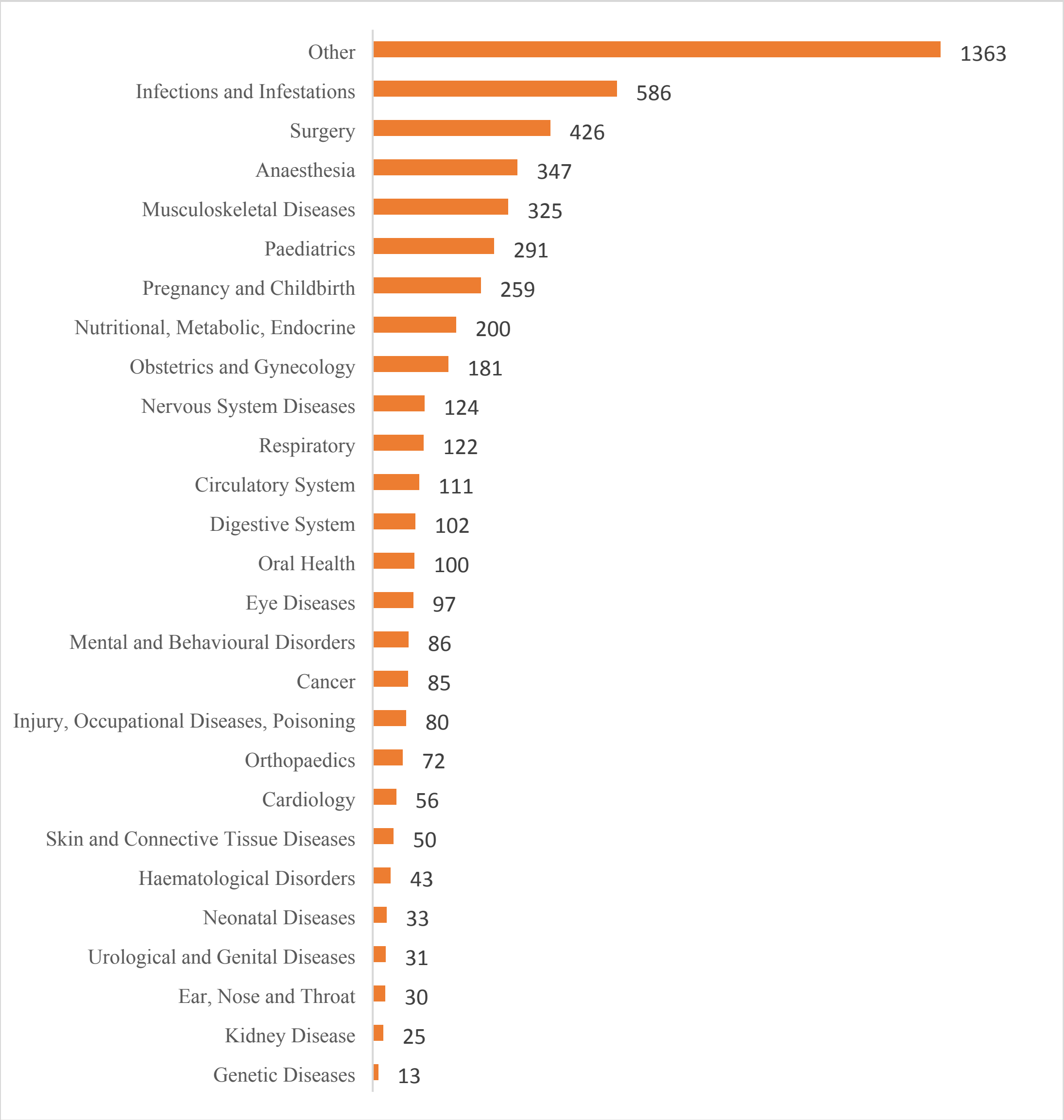

Figure 3: Disease conditions investigated in PACTR registered trials

Among the completed trials (n =1083), most of the records are without results (91%; 989/1083), and less than 10% have results (**figure 4**).

For peer review only - http://bmjopen.bmj.com/site/about/guidelines.xhtml

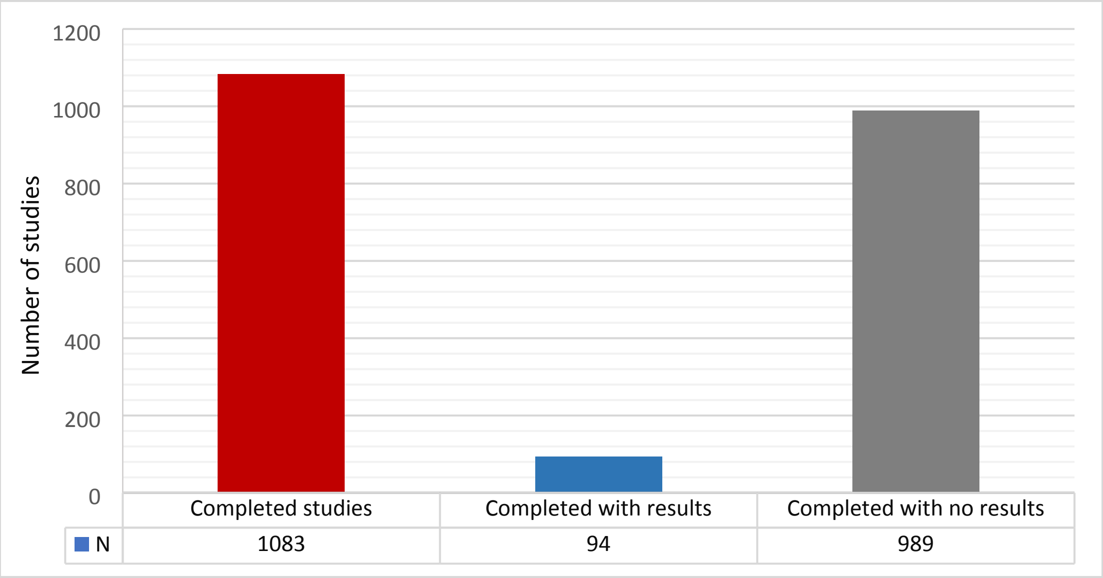

Figure 4 Completed trials with results in PACTR

The reporting section in PACTR was not mandatory until 2019. Our data show that from 2008 until 2017, results reporting was not captured. In 2018, PACTR was relaunched to include the 24-item data set required by ICTRP[12]. The reporting section is the 24th data item which collects information on the plans to share trial data and provides summary results. When PACTR was relaunched in 2018, this field was not mandatory and had options "yes," "no," and "undecided."

peer review only

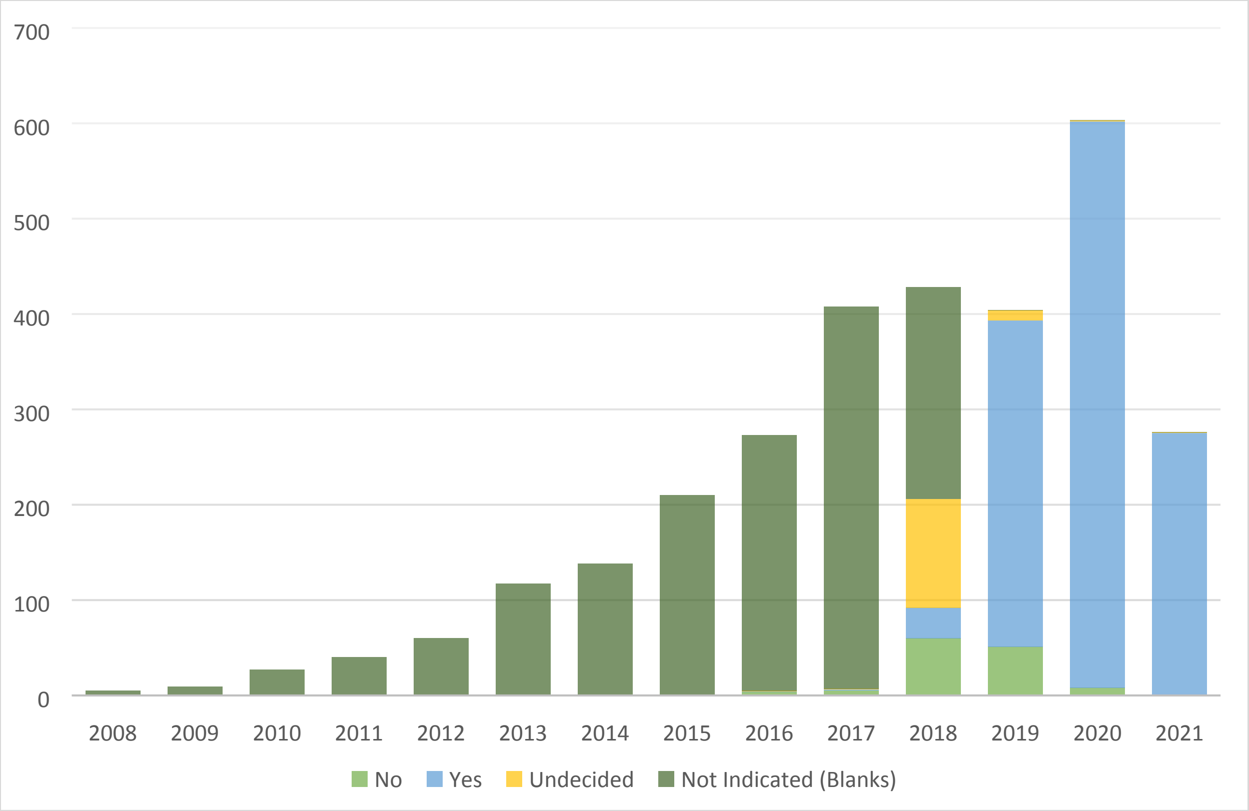

Figure 5: Trends in results reporting over the years

The data shows that 4% (114/2998) of researchers opted to choose undecided when capturing the trial information, while most researchers left this section blank 7% (222/2998). In 2019 when the "undecided option was removed, and the field became mandatory, there was a shift in the trend with 11% (342/2998) indicating "yes" to complete the results reporting section. This trend continues with more trials in 2020 and 2021, opting for completing the reporting section (**figure 5**).

DISCUSSION

For peer review only - http://bmjopen.bmj.com/site/about/guidelines.xhtml

PACTR has seen a growing number of trials registered over the years. We, therefore, report on trends in the registration of clinical trials in PACTR. Understanding the trends will allow for further improvements on the registry and identify issues that the registry team can improve on. Our data shows substantial growth in the number of registered trials over the years. PACTR has seen in 2020 having registered 606 trials contributing to 20% of the analysed trials. Trials are allowed to be registered retrospectively; however, prospective registration of trials is promoted. In our efforts to encourage prospective registration of trials, a slight shift towards prospective trial registration can be seen from 2017.

Prospective trial registration is currently at 55%, while retrospective trial registration is at 45%. Efforts to register trials prospectively are something that needs to be done across all primary registers. Durra et al. 2020 conducted a cross-sectional analysis of published trials registered in registries worldwide and found that prospective registration is deficient [9]. PACTR allows registration of a trial if the researcher indicates when ethics approval has been applied for. We show that among the trials registered in PACTR, 90% have ethics approval which shows that the trials conducted have gone through the ethics approval process. PACTR staff also ensures that the ethics approval is verified to ensure that the data in the registry is correct.

The intervention model in PACTR registered trials indicates that registries may need to adapt to the changing trial designs, as can be seen with the current COVID-19 trials where adaptive trial designs were used [15-17]. Our analysis shows that the most common intervention model was factoral assignment 20%. The studies registered in PACTR show a worrying trend which shows that 55% of the trials have no funding while 23% of the trials are self-funded. Similarly, a recent cross-sectional bibliographic study showed that tuberculosis trials conducted in Africa had a dearth of funding coming from local African governments and NGOs [18]. There should be a shift for

For peer review only - http://bmjopen.bmj.com/site/about/guidelines.xhtml

African governments and funders to create appropriate ways to ensure that total costs of clinical research are provided. Research institutions and universities with a real potential for success should have priority so that resources can be focused on driving research programs for Africa[19].

The other concerning trend from our analysis is that 28% of the trials are listed as not recruiting. This is indicative that researchers do not update the records, which could result in the data being misinterpreted. The "not recruiting" status indicates that participants are still receiving an intervention or being examined, but new participants are not currently recruited or enrolled. However, it may also suggest that this status indicates that all participant visits are completed, but that the study is still open to ethics, data analysis is still ongoing, or the manuscript is pending publication. This suggests a need to build capacity on the 24-item data set [12].

Moreover, capacity building should focus on the important role that a registry serves as a source of data sharing, identifying research gaps, and its essential contribution in the evidence ecosystem[20] rather than another administrative activity to get their trials conducted. Our data show that of the completed trials in the registry, only 3% have results available. This suggests that PACTR needs to partner with funding agencies to ensure that results are in a public domain within a specified period [21], to ensure that reporting of clinical trials is not subjected to selective reporting and publication bias[9, 22, 23].

The most common intervention in the trials conducted in Africa is treatment with drugs (21%), in which the trials registered in PACTR seek to find treatment options for infectious diseases (20%). This explains that in the most common diseases researched in Africa, there is a need for new drugs to curb the pandemics of these diseases.

For peer review only - http://bmjopen.bmj.com/site/about/guidelines.xhtml

For peer review only

The reporting section, item 24, suggests a trend towards being completed to conform to WHO-ICTRP requirements. Results reporting became mandatory in January 2019. Our analysis shows that there has been an improvement with the reporting section being completed. This trend continues with more trials in 2020 and 2021, opting for completing the reporting section. As part of our ongoing analysis, this analysis shows that more needs to be done to build capacity on clinical trials through partnering with regulatory, sponsors, and researchers to ensure that clinical research conducted in Africa meets the global standards.

Limitations

We conducted a descriptive analysis of the data and selected a few fields to analyse what is happening in the registry. We used the ICTRP platform to search for PACTR registered studies, using this approach would exclude other studies because PACTR sends a file monthly while the registry continues to register trials. When analysing the data, the disease category was too vast to understand the specific disease conditions investigated. In future, we will focus on unpacking the disease categories and understand the trends in the diseases being assessed. Also, there are data elements in which a researcher would indicate "other", resulting in many trials with "other" as a disease condition. Assessment of the data allows the PACTR review team to reinforce correct data entry when conducting reviews of the submitted trials and include all mandatory data fields required by WHO.

Conclusion

Registration on PACTR has continued to grow since 2008. PACTR provides valuable data to map clinical trial conduct on the African continent. More work needs to be done to ensure that, as the registry team, we guarantee capacity building in collaboration with the ethics committee, funders,

For peer review only - http://bmjopen.bmj.com/site/about/guidelines.xhtml

and sponsors to provide that PACTR ensure that clinical research conducted in Africa meets the international standards. There is an urgent need to continue to raise awareness for prospective trial registration and reporting of summary results. This will allow researchers to understand the importance of data sharing to contribute to research gaps to find solutions for Africa.

Strengths and limitations of this study

- The Pan African Clinical Trial Register (PACTR) has substantially grown over the years with the number of trials registered.
- The trend analysis allows for opportunities to improve and prioritise activities for PACTR.
- There has been an improvement in the efforts to ensure that the registry collects the 24-data item field after it was made mandatory on 1 January 2019 by the World Health Organization.
- Work needs to be done to encourage researchers to capture the trial results in the registry upon study completion

Reporting patient and public involvement in research

This study is a cross sectional analysis of publicly available clinical trials registered in PACTR. The study's findings will inform how the PACTR registry team will engage with the researchers to promote clinical trial registration.

Funding

The publication cost for this article was paid with funds from the South African Medical Research Council (project code 43500).

For peer review only - http://bmjopen.bmj.com/site/about/guidelines.xhtml

Acknowledgments

The publication cost for this article was paid with funds from the South African Medical Research Council. The views expressed in this article are those of the authors. They do not necessarily reflect the opinions or policies of the South African Medical Research Council, Cochrane, or other institutions that the authors are affiliated with.

Ethics approval

We used data from the Pan African Clinical Trial Registry which stores trials information and make the data publicly available. We conducted analysis of the clinical trials information in the register, therefore ethics approval was required.

Competing interests

The authors declare that they have no competing interests.

Author Contributions

DN conceived and wrote the article's first draft, SN, LM, EP, AH, CSW contributed important intellectual input to subsequent versions of the article. The authors have read and approved the final version of the article for submission.

For peer review only

REFERENCES:

1. Abrams A, Siegfried N: **The Pan African Clinical Trials Registry: year one data analysis of the only African member of the World Health Organization Network of Primary Registries**. *J Evid Based Med* 2010, **3**(4):195-200.
2. Abrams A, Siegfried N: **Growing everyday: the Pan African Clinical Trials Registry**. *J Evid Based Med* 2011, **4**(3):172-178.
3. Abrams AL: **One of a kind--the Pan African Clinical Trials Registry, a regional registry for Africa**. *Pan Afr Med J* 2011, **9**:42.
4. Isaakidis P, Swingler GH, Pienaar E, Volmink J, Ioannidis JP: **Relation between burden of disease and randomised evidence in sub-Saharan Africa: survey of research**. *BMJ* 2002, **324**(7339):702.
5. Abrams A, Siegfried N: **A Pan African Clinical Trials Registry for the specific needs of triallists on the continent**. *S Afr Med J* 2010, **100**(5):294-295.
6. Sim I, Chan AW, Gulmezoglu AM, Evans T, Pang T: **Clinical trial registration: transparency is the watchword**. *Lancet* 2006, **367**(9523):1631-1633.
7. Swingler GH, Pillay V, Pienaar ED, Ioannidis JP: **International collaboration, funding and association with burden of disease in randomized controlled trials in Africa**. *Bull World Health Organ* 2005, **83**(7):511-517.
8. Gulmezoglu AM, Sim I: **International clinical trial registration: any progress?** *Wien Klin Wochenschr* 2006, **118**(1-2):1-2.
9. Al-Durra M, Nolan RP, Seto E, Cafazzo JA: **Prospective registration and reporting of trial number in randomised clinical trials: global cross sectional study of the adoption of ICMJE and Declaration of Helsinki recommendations**. *BMJ* 2020, **369**:m982.
10. De Angelis C, Drazen JM, Frizelle FA, Haug C, Hoey J, Horton R, Kotzin S, Laine C, Marusic A, Overbeke AJ *et al*: **[Clinical trial registration: a statement from the International Committee of Medical Journal Editors]**. *Tidsskrift for den Norske laegeforening : tidsskrift for praktisk medicin, ny raekke* 2004, **124**(18):2339.
11. Zarin DA, Tse T, Williams RJ, Rajakannan T: **Update on Trial Registration 11 Years after the ICMJE Policy Was Established**. *The New England journal of medicine* 2017, **376**(4):383-391.
12. **International Standards for Clinical Trial Registries** [https://apps.who.int/iris/bitstream/handle/10665/274994/9789241514743-eng.pdf]
13. Isaakidis P, Ioannidis JP: **Evaluation of cluster randomized controlled trials in sub-Saharan Africa**. *Am J Epidemiol* 2003, **158**(9):921-926.
14. Swingler GH, Volmink J, Ioannidis JP: **Number of published systematic reviews and global burden of disease: database analysis**. *BMJ* 2003, **327**(7423):1083-1084.
15. Wang SJ: **Recent advances in clinical trial design considerations in Thera"nostics"**. *Contemporary clinical trials* 2020, **96**:106100.
16. Lienhardt C, Nunn A, Chaisson R, Vernon AA, Zignol M, Nahid P, Delaporte E, Kasaeva T: **Advances in clinical trial design: Weaving tomorrow's TB treatments**. *PLoS medicine* 2020, **17**(2):e1003059.
17. Raber-Johnson ML, Gallwitz WE, Sullivan EJ, Storer P: **Innovation in Clinical Trial Design and Product Promotion: Evolving the Patient Perspective With Regulatory and Technological Advances**. *Therapeutic innovation & regulatory science* 2020, **54**(3):519-527.
18. Hohlfeld ASJ, Mathebula L, Pienaar ED, Abrams A, Lutje V, Ndwandwe D, Kredo T: **Tuberculosis treatment intervention trials in Africa: A cross-sectional bibliographic study and spatial analysis**. *PloS one* 2021, **16**(3):e0248621.

For peer review only

For peer review only - http://bmjopen.bmj.com/site/about/guidelines.xhtml

19. Whitworth JA, Kokwaro G, Kinyanjui S, Snewin VA, Tanner M, Walport M, Sewankambo N: **Strengthening capacity for health research in Africa**. *Lancet* 2008, **372**(9649):1590-1593.
20. Vandvik PO, Brandt L: **Future of Evidence Ecosystem Series: Evidence ecosystems and learning health systems: why bother?** *Journal of clinical epidemiology* 2020, **123**:166-170.
21. Knowles RL, Ha KP, Mueller J, Rawle F, Parker R: **Challenges for funders in monitoring compliance with policies on clinical trials registration and reporting: analysis of funding and registry data in the UK**. *BMJ open* 2020, **10**(2):e035283.
22. Edem B, Onwuchekwa C, Wariri O, Nkereuwem E, Nkereuwem OO, Williams V: **Trends in clinical trial registration in sub-Saharan Africa between 2010 and 2020: a cross-sectional review of three clinical trial registries**. *Trials* 2021, **22**(1):472.
23. Hart B, Lundh A, Bero L: **Effect of reporting bias on meta-analyses of drug trials: reanalysis of meta-analyses**. *BMJ* 2012, **344**:d7202.

For peer review only

For peer review only - http://bmjopen.bmj.com/site/about/guidelines.xhtml

BMJ Open

Practices and trends in clinical trial registration in the Pan African Clinical Trials Registry (PACTR): a descriptive analysis of registration data

Journal:	*BMJ Open*
Manuscript ID	bmjopen-2021-057474.R1
Article Type:	Original research
Date Submitted by the Author:	29-Nov-2021

Complete List of Authors:	Ndwandwe, Duduzile; South African Medical Research Council<br>Runeyi, Sinazo; South African Medical Research Council, Cochrane South Africa<br>Pienaar, Elizabeth ; South African Medical Research Council, Cochrane South Africa<br>Mathebula, Lindi; South African Medical Research Council, Cochrane SA<br>Hohlfeld, Ameer ; South African Medical Research Council, Cohrane South Africa<br>Wiysonge, Charles; South African Medical Research Council, Cochrane South Africa

**Primary Subject Heading**:	Public health
Secondary Subject Heading:	Global health, Health policy, Evidence based practice, Epidemiology, Ethics
Keywords:	Clinical trials < THERAPEUTICS, PUBLIC HEALTH, Public health < INFECTIOUS DISEASES

SCHOLARONE™
Manuscripts

I, the Submitting Author has the right to grant and does grant on behalf of all authors of the Work (as defined in the below author licence), an exclusive licence and/or a non-exclusive licence for contributions from authors who are: i) UK Crown employees; ii) where BMJ has agreed a CC-BY licence shall apply, and/or iii) in accordance with the terms applicable for US Federal Government officers or employees acting as part of their official duties; on a worldwide, perpetual, irrevocable, royalty-free basis to BMJ Publishing Group Ltd ("BMJ") its licensees and where the relevant Journal is co-owned by BMJ to the co-owners of the Journal, to publish the Work in this journal and any other BMJ products and to exploit all rights, as set out in our licence.

The Submitting Author accepts and understands that any supply made under these terms is made by BMJ to the Submitting Author unless you are acting as an employee on behalf of your employer or a postgraduate student of an affiliated institution which is paying any applicable article publishing charge ("APC") for Open Access articles. Where the Submitting Author wishes to make the Work available on an Open Access basis (and intends to pay the relevant APC), the terms of reuse of such Open Access shall be governed by a Creative Commons licence – details of these licences and which Creative Commons licence will apply to this Work are set out in our licence referred to above.

Other than as permitted in any relevant BMJ Author's Self Archiving Policies, I confirm this Work has not been accepted for publication elsewhere, is not being considered for publication elsewhere and does not duplicate material already published. I confirm all authors consent to publication of this Work and authorise the granting of this licence.

For peer review only

Practices and trends in clinical trial registration in the Pan African Clinical Trials Registry (PACTR): a descriptive analysis of registration data

Duduzile Ndwandwe[1*]; Sinazo Runeyi[1]; Elizabeth D Pienaar[1]; Lindi Mathebula[1]; Ameer Hohlfeld[1]; Charles S. Wiysonge[1,2,3]

[1]Cochrane South Africa, South African Medical Research Council, Tygerberg, South Africa

[2]Division of Epidemiology and Biostatistics, Faculty of Medicine and Health Sciences, Stellenbosch University, Tygerberg, 7505, Cape Town, South Africa.

[3]School of Public Health and Family Medicine, Faculty of Health Sciences, University of Cape Town, Observatory, 7925, Cape Town, South Africa.

*Correspondence author: Duduzile Ndwandwe, Cochrane South Africa, South African Medical Research Council, Tygerberg, South Africa. Email: Duduzile.Ndwandwe@mrc.ac.za

For peer review only - http://bmjopen.bmj.com/site/about/guidelines.xhtml

Abstract

Background

The Pan African Clinical Trials Register (PACTR) is a World Health Organization (WHO) International Clinical Trials Registry Platform (ICTRP) primary register, which caters for clinical trials conducted in Africa. PACTR is the first and, at present, the only member of the Network of WHO Primary Registers in Africa. The aim is to describe and report on the trends of trial records registered in PACTR.

Methods

PACTR was established in 2007 as the AIDS, Tuberculosis, and Malaria (ATM) Clinical Trials Registry. The scope of the registry was then expanded in 2009 to include all diseases. This is a cross-sectional study of trials registered in PACTR from inception to 18 August 2021. A descriptive analysis of the use and trends of the following data fields: study intervention, disease condition, sex of the participants, sample size, ethics, funding, and availability of results conducted using Microsoft Excel.

Results

The number of trials registered has increased year on year, reaching 606 trials registered in 2020. The total number of trials registered at the time of the analysis was 2998. More than half of the trials in the registry (1655/2998 i.e. 55%) were prospectively registered. Ethical approval was received by 90% (2691/2998) of the registered trials. Factorial assignment as an intervention model was in 20% (589/2998) of the trials registered. There were 36% (1083/2998) completed trials, of which 3% (94/1083) had results available in the registry. The most dominant funding source indicated was self-funding in 23% (693/2998) of the registered trials, and 55% (1639/2998) had no funding.

For peer review only

For peer review only - http://bmjopen.bmj.com/site/about/guidelines.xhtml

Conclusion

Registration on PACTR continues to grow; however, our analysis shows that researchers' capacity-building is needed to understand the importance of the registry and how this information informs healthcare decisions. Promoting prospective trial registration remains critical to avoid selective reporting bias to inform research gaps.

Keywords: Pan African Clinical Trial Register, Clinical trial registration, Prospective trial registration

For peer review only

For peer review only - http://bmjopen.bmj.com/site/about/guidelines.xhtml

Strengths and limitations of this study

- We provided a comprehensive descriptive assessment of the trials registered in the Pan African Clinical Trial Register (PACTR)
- We conducted a descriptive analysis to assess the trends of the fields collected in the registry records to improve and prioritise activities for PACTR administration staff.
- We selected mandatory data fields to analyse to precisely assess the general trends in the trial records without analysing the free-text data captured
- There were some unavoidable missing data and variations for certain data fields, which might bias the results.

For peer review only

Introduction

The Pan African Clinical Trials Registry (PACTR) (www.pactr.org) was established from the AIDS, Tuberculosis, and Malaria Registry based at the South African Cochrane Centre [1-3]. The registry was established with Cochrane's Infectious Diseases Group (CIDG), based at the Liverpool School of Tropical Medicine and the World Health Organization (WHO). PACTR is the only African member of the WHO Network of Primary Registers, which transfers trial information to the WHO International Clinical Trials Registry Platform (WHO-ICTRP) (https://www.who.int/clinical-trials-registry-platform) every month [4, 5]. WHO-ICTRP serves as a platform aligned with the International Committee of Medical Journal Editors (ICMJE) for prospective trial registration. PACTR contributes to regional transparency and harmonisation of clinical trial research [6, 7]. A database contains essential administrative and scientific information about planned, ongoing, and completed trials in a clinical trials registry [6-8]. Thus, registration of all interventional trials is considered scientific, ethical, and responsible [9-11]. Accessing clinical trials information allows informing decision-making on healthcare decisions based on all available evidence [9]. Such decisions cannot be easily made if publication bias and selective reporting exist [9].

Furthermore, the Declaration of Helsinki indicates that "Every clinical trial must be registered in a publicly accessible database before recruitment of the first subject" [9]. In the case of clinical trials, before the first participant is recruited, the information on the trial must be captured in a publicly accessible database unless the sponsor or researcher has permission to delay this to a later stage. Trial registration is one of the efforts being made to ensure transparency in clinical research, accessible patient data for subsequent analysis, and publication of results irrespective of the trial

For peer review only - http://bmjopen.bmj.com/site/about/guidelines.xhtml

outcome. This further allows for decisions related to the safety and efficacy of drugs, vaccines, and medical devices in humans, supported by the best available scientific evidence.

This, therefore, imply that clinical trial registration should advocate for prospective trial registration and that all registered trials publish their findings[12]. Trial registration further supports evidence-based medical practice, which heavily relies on available data in the public domain so that informed healthcare decisions can be made[13]. Bringing in data from clinical trials within reach of clinicians, regulators, and external stakeholders enhances the clinical trial data[13]. Prospective trial registration and subsequent results reporting are global efforts to ensure complete research transparency. Clinical trials may be registered without ethics approval, provided that recruitment of study participants has not commenced. Even Journal editors, ethics committees/institutional review boards (IRBs), regulatory authorities, and funding agencies all support the call for research transparency requiring trials to be prospectively registered[14].

There has been a push from governments and international organisations, especially since 2005, to make clinical trial information more widely available and standardise registries and registering processes. The World Health Organization (WHO) has published international Standards for Clinical Trial Registries to achieve consensus on both the minimal and the optimal operating standards for trial registration [14]. To adhere to WHO practices that ensure that collected data are not duplicated and provide meaningful information, registry staff scrutinise each application and perform regular quality checks to ensure quality data is contained in the registry.

A further benefit to registering trials prospectively in a registry is that it allows for similar or identical trials to be known, making it possible for researchers and funding agencies to avoid unnecessary duplication [7]. Also, describing clinical trials in progress makes it easier to identify research gaps for new research to advance the knowledge gaps. Registries provide quality checks

For peer review only - http://bmjopen.bmj.com/site/about/guidelines.xhtml

on the data submitted as part of the registration process, leading to improvements in the quality of clinical trials publicly available and pointing out potential problems early in the research design to improve clinical research conducted.

Although in the past, research on the clinical trial landscape provided key insights into the global burden of disease, and more generally, the global and regional clinical trial landscapes [4, 7, 15, 16], before PACTR, there was no regional support for longitudinal monitoring of planned and ongoing African clinical trials. PACTR is unique in recognising that African researchers face additional challenges in trial registration and seeks to provide feasible ways of overcoming these barriers [2]. PACTR has seen substantial growth in the number of trials registered from inception until recently. In this cross-sectional survey of the PACTR database, we report on the trends in the clinical trials registered.

MATERIALS AND METHODS

This was a descriptive analysis of the trends in clinical trials registered in the Pan African Clinical Trial Register (PACTR), www.pactr.org.

Data description and source

We used the WHO ICTRP, https://www.who.int/clinical-trials-registry-platform, a registry platform collating information from registries across the globe to be a one-stop portal to access clinical trial records[17]. The study used the WHO definition of a clinical trial: "any research study that prospectively assigns human participants or groups of humans to one or more health-related interventions to evaluate the effects on health outcomes"[14]. We used the advanced search function of ICTRP to identify these clinical trials registered in the PACTR on 18 August 2021.

For peer review only

Data management and analysis

Data were downloaded from WHO-ICTRP one researcher (SR) on 18 August 2021 and exported into an excel spreadsheet. All records were quality-checked by a second researcher (DN). In each record, the following data items were used for analysis: registration status, disease condition, sex of the participants in the trials; sponsor, intervention type, funding source, the age range of participants, intervention model, phase of the trial, and overall status. We conducted a descriptive analysis of the use and trends of the registered trials in PACTR to understand the pattern of trial registration over the years using Microsoft Excel.

Results

We report on the trends for trials registered in PACTR appearing in the ICTRP portal. PACTR is one of the WHO primary registers which sends data monthly to the ICTRP for one-stop to access trials records. We downloaded from the ICTRP on 18 August 2021. We used the search output to only select trials from the PACTR registry. A total of 2998 trial records were retrieved and used for analysis.

PACTR has grown substantially since its inception, with each year showing a steady increase in the number of trials registered. The year 2020 had the most registered trials (n=606). We anticipate that this increase will be seen even in 2021 (**figure 1**). **Insert Figure 1 here**

We further extrapolated the trials registered in 2020 to assess whether the significant increase was because of the COVID-19 pandemic, which has seen a rise in research activity.

Insert Figure 2 here

We found that 7% (42/606) were COVID related trials in the year 2020 and among these trials, 46% were on treatment and 20% on vaccines (figure 2).

For peer review only - http://bmjopen.bmj.com/site/about/guidelines.xhtml

Table 1 shows our analysis of some of the registry data items. Generally, there has been an increase in the number of trials registered in PACTR, with n= 2998 identified at the analysis time (figure1). There are 1083 (36%) completed trials, with 94 (3%) having results available in the registry. Twenty-eight percent (28%) of trials registered (836/2998) are listed as not recruiting, while 25% (755/2998) are recruiting participants. 55% (1655/2998) of the trials are registered prospectively, with the remaining 45% (1343/2998) registered retrospectively.

Our data show that most of the trials registered in PACTR have ethics approval (2691/2998, i.e. 90%). The intervention model refers to the general design of the strategy for assigning therapies or interventions being investigated to participants in a clinical study. Types of intervention models include single group assignment, parallel assignment, cross-over assignment, and factorial assignment. The most common intervention model in the registered trials was factorial assignment (589/2998, i.e. 20%), which means that the trial would have two (or more) intervention comparisons carried out simultaneously. The trial phases show an almost equal distribution for all clinical trial phases. We assessed the sponsor of the registered trials and reported that the sponsor could be the funder. Our data show that 55% (1639/2998) of the trials have no funding, while 23% (693/2998) are self-funded. Many trials (2240/2998, i.e. 75%) recruited both male and female participants. The median sample size was 1140.7 participants, with rannging from 0 to 1,087,000.

For peer review only

For peer review only - http://bmjopen.bmj.com/site/about/guidelines.xhtml

175 **Table 1: Characteristics of trials in PACTR**

**Description**	***N* (%)**
**Number of trials registered**	2998
**Number of studies completed**	1083 (36.1)
**Number of completed with results**	94 (3.1)
**Overall trial status**	
Completed	1083 (36.1)
Recruiting	755 (25.2)
Not yet recruiting/pending	836 (27.9)
Recruiting	755 (25.2)
Stopped/terminated	-
Suspended	7 (0.2)
Pending	836 (27.5)
Other/unknown	317 (10.6)
**Prospective/retrospective**	
Prospectively registered	1655 (55.2)
Retrospectively registered	1343 (44.8)
**Intervention model**	
Parallel assignment	2124 (70.8)
Single group assignment	59 (2.0)
Cross-over assignment	201 (6.7)
Factorial assignment	589 (19.6)
Sequential assignment	13 (0.4)
None (open label)	12 (0.4)
**Phase**	
Not reported	2310 (77.1)
Phase I	192 (6.5)

For peer review only - http://bmjopen.bmj.com/site/about/guidelines.xhtml

Phase II	138 (4.6)
Phase III	199 (6.6)
Phase IV	155 (5.2)
**Primary Sponsor**	
University	196 (6.5)
Industry or non-governmental organisation	61 (2.0)
Government	107 (3.6)
Charities	94 (3.1)
Hospital	67 (2.2)
Self-funded	693 (23.1)
Funding agency	142 (4.7)
Other	45 (1.5)
No funding	1639 (54.7)
**Ethics approval received**	
Yes	2691 (90)
No	307 (10)
**Sex**	
Both males and females	2240 (74.7)
Female	628 (20.9)
Male	130 (4.3)
**The target number of participants**	
Minimum, maximum	0- 1, 087, 000
Mean (standard deviation)	1, 140.7 (26, 156.8)
Median (IQR)	80 (1-125)

For peer review only

Researchers have an option in the PACTR registry to indicate the type of intervention for the trial. We show that most trials use drug treatment (622/2998, i.e 21%) as an intervention (**figure 3**).

Insert Figure 3 here

The most common disease conditions investigated in the trials conducted in PACTR registered trials were infections and infestations with 20% (586/2998), followed by the surgery category with 14% (426/2998) trials. The trials listed surgery as a disease condition included any intervention in a trial where medical and surgical care was provided. Such studies focus on diseases, injuries, and conditions affecting the abdomen, breasts, digestive system, endocrine system, and skin. Also, these trials evaluate biopsies, lab tests, and imaging tests as part of delivering care (**Figure 4**).

Insert Figure 4 here

Among the completed trials (n =1083), most of the records are without results (91%; 989/1083), and less than 10% have results (**figure 5**).

Insert Figure 5 here

The reporting section in PACTR was not mandatory until 2019. Our data show that from 2008 until 2017, results reporting was not captured. In 2018, PACTR was relaunched to include the 24-item data set required by ICTRP[14]. The reporting section is the 24th data item which collects information on the plans to share trial data and provides summary results. When PACTR was relaunched in 2018, this field was not mandatory and had options "yes," "no," and "undecided." We, therefore, assessed whether the trials in PACTR reflected the occurrence of adding the additional data fields.

For peer review only - http://bmjopen.bmj.com/site/about/guidelines.xhtml

Insert Figure 6 here

The data shows that 4% (114/2998) of researchers opted to choose undecided when capturing the trial information, while most researchers left this section blank 7% (222/2998). In 2019 when the "undecided option was removed, and the field became mandatory, there was a shift in the trend with 11% (342/2998) indicating "yes" to complete the results reporting section. This trend continues with more trials in 2020 and 2021, opting for completing the reporting section (**figure 6**).

DISCUSSION

PACTR has seen a growing number of trials registered over the years. We, therefore, report on trends in the registration of clinical trials in PACTR. Understanding the trends will allow for further improvements on the registry and identify issues that the registry team can improve on. Our data shows substantial growth in the number of registered trials over the years. PACTR saw in 2020 had registered 606 trials contributing to 20% of the analysed trials. COVID-19 related trials only contributed to 7% of the increasing trials registered in 2020. Among these trials, 46% (19/41) being investigated, the most dominant intervention was treatment for COVID-19. This trend is on an upward trajectory as even in the year 2021, there are 15 trials related to COVID-19[18].

In our efforts to encourage prospective registration of trials, a slight shift towards prospective trial registration can be seen from 2017. Prospective trial registration is currently at 55%, while retrospective trial registration is 45% (Figure 1). Trials can be registered retrospectively; however, the prospective registration of trials is encouraged to ensure transparency in research conduct, thus

For peer review only - http://bmjopen.bmj.com/site/about/guidelines.xhtml

reducing publication and reporting bias[9]. Efforts to register trials prospectively need to be done across all primary registers. Durra et al. 2020 conducted a cross-sectional analysis of published trials registered in registries worldwide and found that prospective registration is deficient [9]. PACTR allows a trial registration if the researcher indicates when ethics approval has been applied for. We show that among the trials registered in PACTR, 90% have ethics approval which shows that the trials conducted have gone through the ethics approval process. PACTR staff also ensures that the ethics approval is verified to ensure that the data in the registry is correct.

The intervention model in PACTR registered trials indicates that registries may need to adapt to the changing trial designs, as seen with the current COVID-19 trials where adaptive trial designs were used [19-21]. Our analysis shows that the most common intervention model was factorial assignment 20%. The studies registered in PACTR show a worrying trend which shows that 55% of the trials have no funding while 23% of the trials are self-funded. Similarly, a recent cross-sectional bibliographic study showed that tuberculosis trials conducted in Africa had a dearth of financing for local African governments and NGOs [22]. There should be a shift for African governments and funders to create appropriate ways to ensure that total costs of clinical research are provided. Research institutions and universities with a real potential for success should have priority so that resources can be focused on driving research programs for Africa[23].

The other concerning trend from our analysis is that 28% of the trials are listed as not recruiting. This is indicative that researchers do not update the records, which could result in the data being misinterpreted. The "not recruiting" status indicates that participants are still receiving an intervention or being examined, but new participants are not currently recruited or enrolled. However, it may also suggest that this status indicates that all participant visits are completed. The

For peer review only - http://bmjopen.bmj.com/site/about/guidelines.xhtml

study is still open to ethics, data analysis is still ongoing, or the manuscript is pending publication. This suggests a need to build capacity on the 24-item data set [14].

Moreover, capacity building should focus on the vital role of registries as a source of data sharing, identifying research gaps, and its essential contribution in the evidence ecosystem[23] rather than another administrative activity to conduct their trials. Our data show that of the completed trials in the registry, only 3% have results available. This suggests that PACTR needs to partner with funding agencies to ensure that results are in a public domain within a specified period [24] and that clinical trial reporting is not subjected to selective reporting and publication bias[9, 25, 26].

The most common intervention in the trials conducted in Africa is treatment with drugs (21%), in which the trials registered in PACTR seek to find treatment options for infectious diseases (20%). This explains that in the most common diseases researched in Africa, there is a need for new drugs to curb the pandemics of these diseases.

The reporting section, item 24, suggests a trend towards being completed to conform to WHO-ICTRP requirements. Results reporting became mandatory in January 2019. Our analysis indicated an improvement in the reporting section completed. This trend continues with more trials in 2020 and 2021, opting for completing the reporting section. As part of our ongoing analysis, this analysis shows that more needs to be done to build capacity on clinical trials through partnering with regulatory, sponsors, and researchers to ensure that clinical research conducted in Africa meets the global standards.

Limitations

We conducted a descriptive analysis of the data to analyse what is happening in the registry. We used the ICTRP platform to search for PACTR registered studies. This approach may have

For peer review only

excluded trials currently being processed at the time of downloading this data file which may be prior to sending montly data file to ICTRP, thus underrepresenting the total number of registered trials. The disease category was too vast to understand the specific disease conditions investigated when analyzing the data. In the future, we will focus on unpacking the disease categories and understanding the trends in the diseases being evaluated. Also, there are data elements in which a researcher would indicate "other," resulting in many trials with "other" as a disease condition. Assessment of the data allows the PACTR review team to reinforce correct data entry when conducting reviews of the submitted trials and include all mandatory data fields required by WHO. Our analysis did not have the free text data captured in the registry, which will need further unpacking to understand the trends of trial registration. The description of a sponsor into categories may limit what the researcher identifies as a sponsor to how we classified the sponsors leading to some variation.

Conclusion

Registration on PACTR has continued to grow since 2008. PACTR provides valuable data to map clinical trial conduct on the African continent. More work needs to be done to ensure that, as the registry team, we guarantee capacity building in collaboration with the ethics committee, funders, and sponsors to provide that PACTR ensures that clinical research conducted in Africa meets international standards. There is an urgent need to continue to raise awareness for prospective trial registration and reporting of summary results. This will allow researchers to understand the importance of data sharing to contribute to research gaps to find solutions for Africa.

For peer review only - http://bmjopen.bmj.com/site/about/guidelines.xhtml

Patient and public involvement

No patient or public involvement.

Funding

The publication cost for this article is supported with funds from the South African Medical Research Council (project code 43500).

Acknowledgments

The authors acknowledges the South African Medical Research Council for funding support. The views expressed in this article are those of the authors. They do not necessarily reflect the opinions or policies of the South African Medical Research Council, Cochrane, or other institutions that the authors are affiliated with.

Ethics approval We conducted an analysis of publicly available clinical trials information. Therefore ethics approval was not required.

Competing interests

The authors declare that they have no competing interests.

Author Contributions

DN wrote the first draft, coordinated and integrated comments from co-authors, approved the final version for publication, and is the guarantor of the manuscript. SN and LM conducted the analysis of the data. SN, LM, EP, AH, CSW critically revised successive drafts of the manuscript, provided

For peer review only

important intellectual input, and approved the final version of the manuscript. The authors have read and approved the article's final version for submission.

Data availability statement.

The data underlying the study will be made available to other researchers upon request. A file of the data will be shared. Additionally, the publication will be shared on the PACTR registry database and shared with the WHO ICTRP.

REFERENCES:

1. Abrams A, Siegfried N: **The Pan African Clinical Trials Registry: year one data analysis of the only African member of the World Health Organization Network of Primary Registries**. *J Evid Based Med* 2010, **3**(4):195-200.
2. Abrams A, Siegfried N: **Growing everyday: the Pan African Clinical Trials Registry**. *J Evid Based Med* 2011, **4**(3):172-178.
3. Abrams AL: **One of a kind--the Pan African Clinical Trials Registry, a regional registry for Africa**. *Pan Afr Med J* 2011, **9**:42.
4. Isaakidis P, Swingler GH, Pienaar E, Volmink J, Ioannidis JP: **Relation between burden of disease and randomised evidence in sub-Saharan Africa: survey of research**. *BMJ* 2002, **324**(7339):702.
5. Abrams A, Siegfried N: **A Pan African Clinical Trials Registry for the specific needs of triallists on the continent**. *S Afr Med J* 2010, **100**(5):294-295.
6. Sim I, Chan AW, Gulmezoglu AM, Evans T, Pang T: **Clinical trial registration: transparency is the watchword**. *Lancet* 2006, **367**(9523):1631-1633.
7. Swingler GH, Pillay V, Pienaar ED, Ioannidis JP: **International collaboration, funding and association with burden of disease in randomized controlled trials in Africa**. *Bull World Health Organ* 2005, **83**(7):511-517.
8. Gulmezoglu AM, Sim I: **International clinical trial registration: any progress?** *Wien Klin Wochenschr* 2006, **118**(1-2):1-2.
9. Al-Durra M, Nolan RP, Seto E, Cafazzo JA: **Prospective registration and reporting of trial number in randomised clinical trials: global cross sectional study of the adoption of ICMJE and Declaration of Helsinki recommendations**. *BMJ* 2020, **369**:m982.
10. De Angelis C, Drazen JM, Frizelle FA, Haug C, Hoey J, Horton R, Kotzin S, Laine C, Marusic A, Overbeke AJ *et al*: **[Clinical trial registration: a statement from the International Committee of Medical Journal Editors]**. *Tidsskrift for den Norske laegeforening : tidsskrift for praktisk medicin, ny raekke* 2004, **124**(18):2339.
11. Zarin DA, Tse T, Williams RJ, Rajakannan T: **Update on Trial Registration 11 Years after the ICMJE Policy Was Established**. *The New England journal of medicine* 2017, **376**(4):383-391.

For peer review only - http://bmjopen.bmj.com/site/about/guidelines.xhtml

For peer review only

12. Authority HR: **Research Transparency** In. Edited by Authority NR. 2 Redman Place, Stratford, London, E20 1JQ; 2020.
13. Joshi M, Bhardwaj P: **Impact of data transparency: Scientific publications**. *Perspectives in clinical research* 2018, **9**(1):31-36.
14. **International Standards for Clinical Trial Registries** [https://apps.who.int/iris/bitstream/handle/10665/274994/9789241514743-eng.pdf]
15. Isaakidis P, Ioannidis JP: **Evaluation of cluster randomized controlled trials in sub-Saharan Africa**. *Am J Epidemiol* 2003, **158**(9):921-926.
16. Swingler GH, Volmink J, Ioannidis JP: **Number of published systematic reviews and global burden of disease: database analysis**. *BMJ* 2003, **327**(7423):1083-1084.
17. Ghersi D, Pang T: **En route to international clinical trial transparency**. *Lancet* 2008, **372**(9649):1531-1532.
18. Tsanni A: **African scientists race to test COVID drugs - but face major hurdles**. In: *Nature* vol. 599; 2021: 25-27
19. Wang SJ: **Recent advances in clinical trial design considerations in Thera"nostics"**. *Contemporary clinical trials* 2020, **96**:106100.
20. Lienhardt C, Nunn A, Chaisson R, Vernon AA, Zignol M, Nahid P, Delaporte E, Kasaeva T: **Advances in clinical trial design: Weaving tomorrow's TB treatments**. *PLoS medicine* 2020, **17**(2):e1003059.
21. Raber-Johnson ML, Gallwitz WE, Sullivan EJ, Storer P: **Innovation in Clinical Trial Design and Product Promotion: Evolving the Patient Perspective With Regulatory and Technological Advances**. *Therapeutic innovation & regulatory science* 2020, **54**(3):519-527.
22. Hohlfeld ASJ, Mathebula L, Pienaar ED, Abrams A, Lutje V, Ndwandwe D, Kredo T: **Tuberculosis treatment intervention trials in Africa: A cross-sectional bibliographic study and spatial analysis**. *PloS one* 2021, **16**(3):e0248621.
23. Whitworth JA, Kokwaro G, Kinyanjui S, Snewin VA, Tanner M, Walport M, Sewankambo N: **Strengthening capacity for health research in Africa**. *Lancet* 2008, **372**(9649):1590-1593.
24. Knowles RL, Ha KP, Mueller J, Rawle F, Parker R: **Challenges for funders in monitoring compliance with policies on clinical trials registration and reporting: analysis of funding and registry data in the UK**. *BMJ open* 2020, **10**(2):e035283.
25. Edem B, Onwuchekwa C, Wariri O, Nkereuwem E, Nkereuwem OO, Williams V: **Trends in clinical trial registration in sub-Saharan Africa between 2010 and 2020: a cross-sectional review of three clinical trial registries**. *Trials* 2021, **22**(1):472.
26. Hart B, Lundh A, Bero L: **Effect of reporting bias on meta-analyses of drug trials: reanalysis of meta-analyses**. *BMJ* 2012, **344**:d7202.

For peer review only - http://bmjopen.bmj.com/site/about/guidelines.xhtml

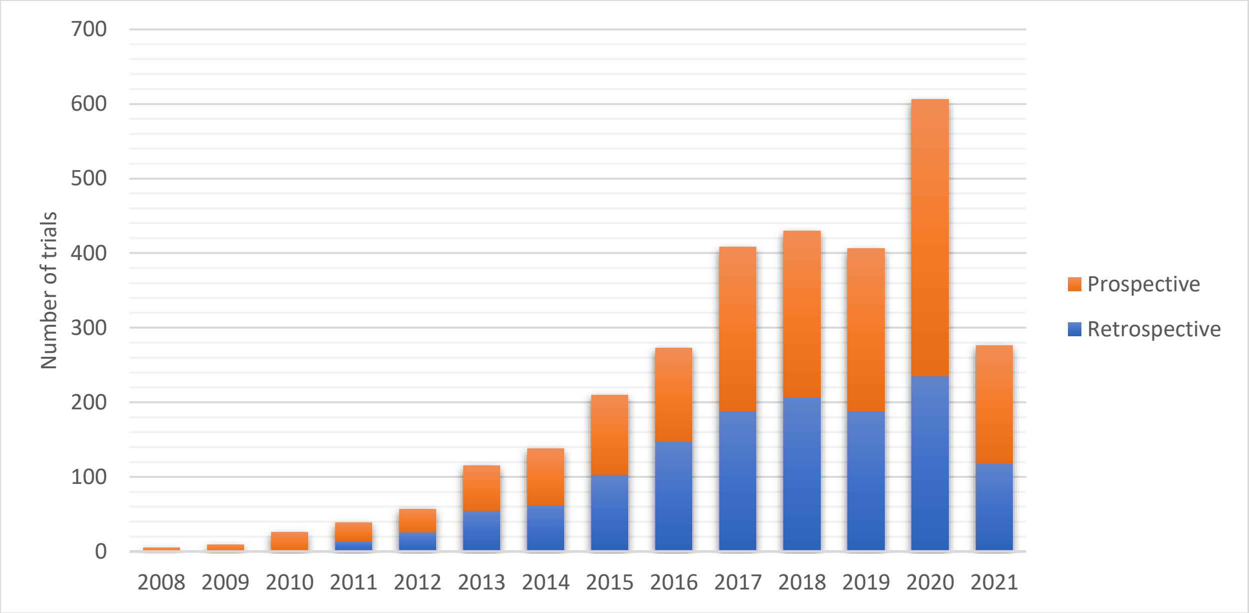

Figure 1: Number of retrospective and prospective registrations on PACTR by year

For peer review only

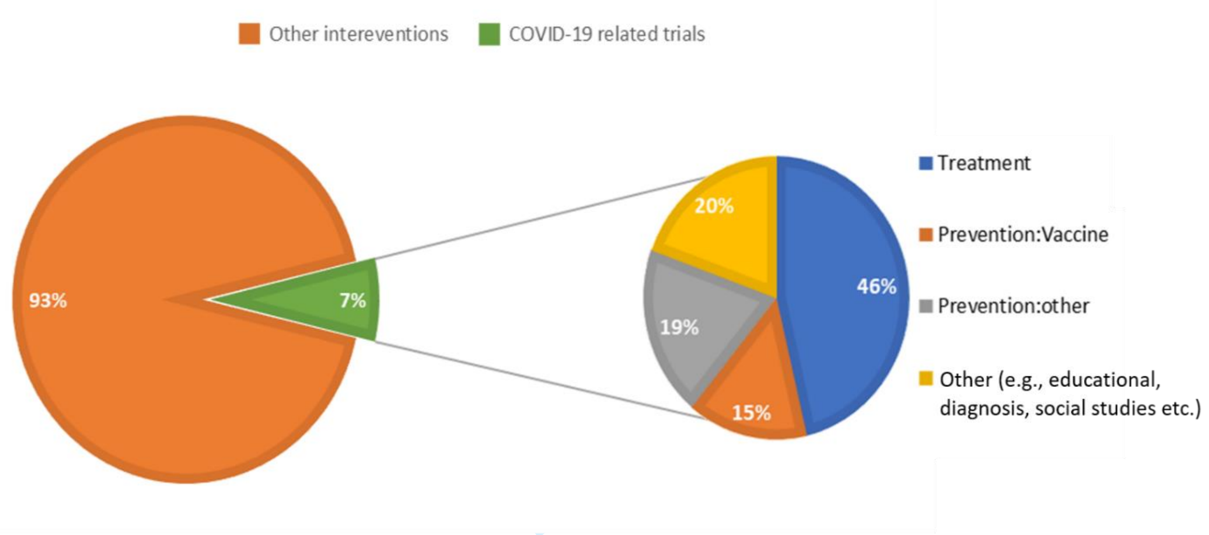

Figure 2: An assessment of the trials registered in the year 2020.

For peer review only

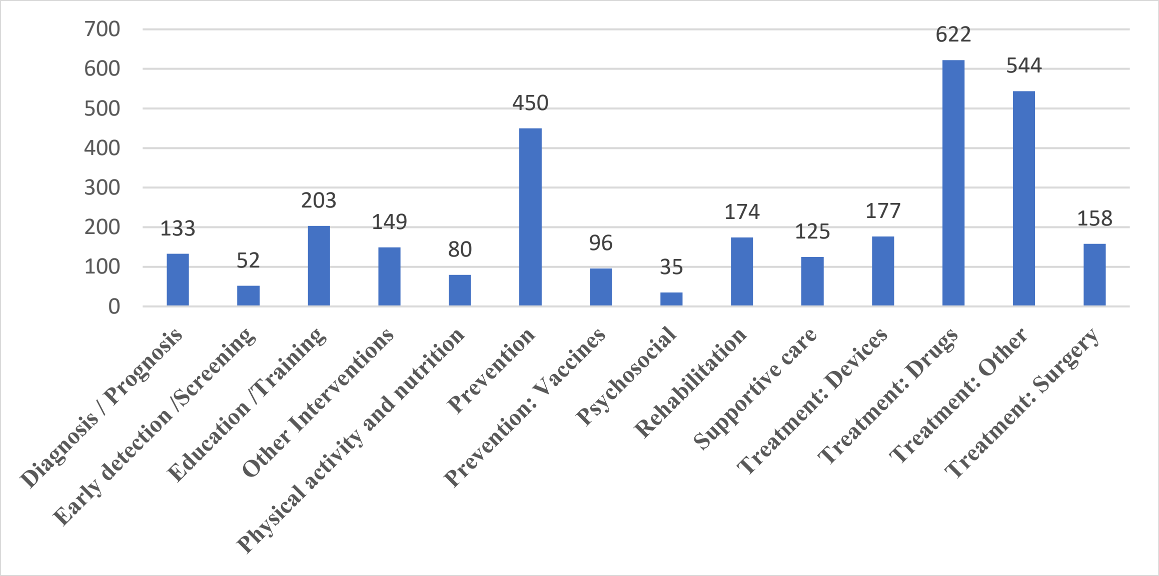

Figure 3: Type of intervention used for the registered trials in PACTR

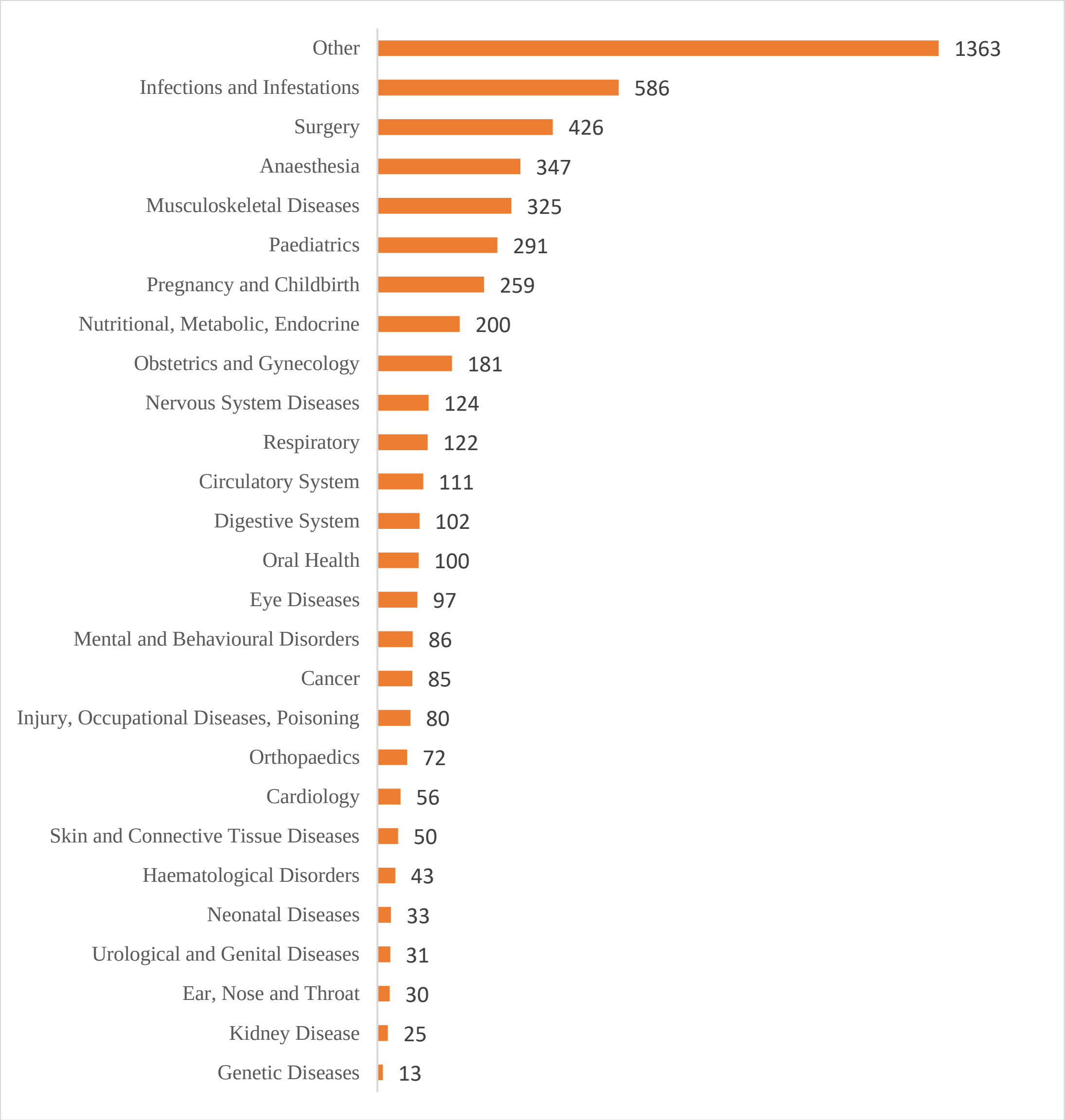

Figure 4: Disease conditions investigated in PACTR registered trials

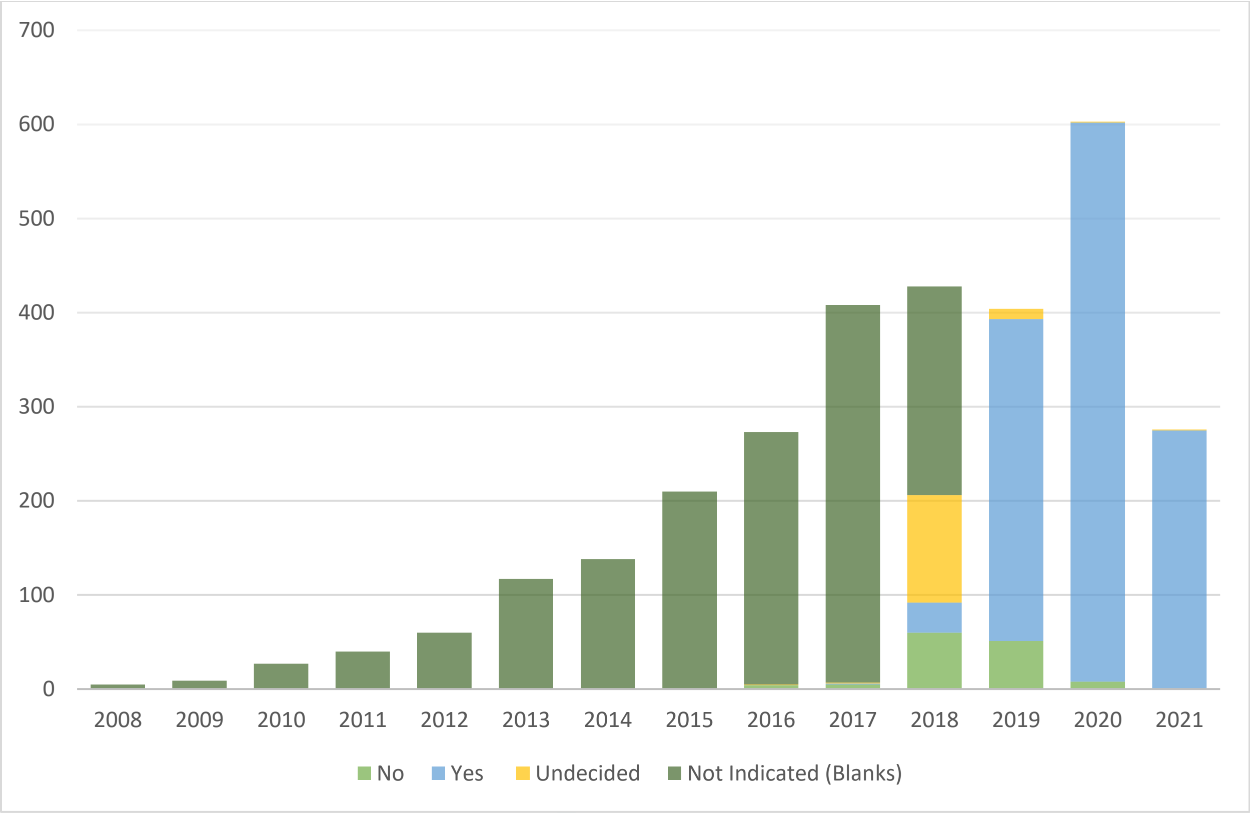

Figure 6: Trends in results reporting over the years

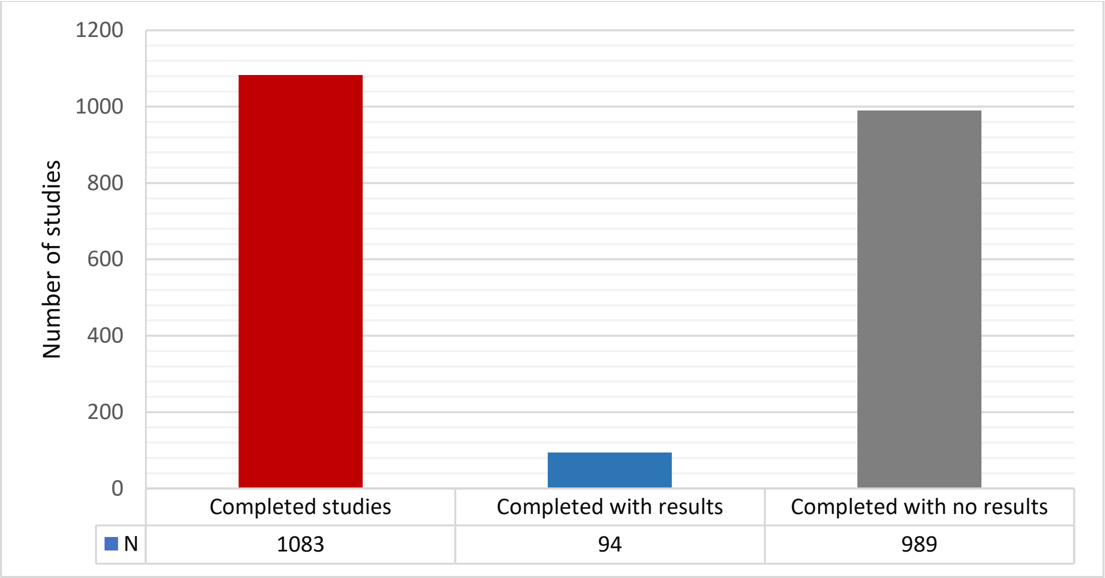

Figure 5: Completed trials with results in PACTR

peer review only

Rotavirus vaccine clinical trials: a cross sectional analysis of clinical trials registries

STROBE Statement—Checklist of items that should be included in reports of ***cross-sectional studies***

	Item No	Recommendation	Page No
**Title and abstract**	1	(*a*) Indicate the study's design with a commonly used term in the title or the abstract	1
		(*b*) Provide in the abstract an informative and balanced summary of what was done and what was found	2-3
**Introduction**			
Background/rationale	2	Explain the scientific background and rationale for the investigation being reported	4-6
Objectives	3	State specific objectives, including any prespecified hypotheses	6
**Methods**			
Study design	4	Present key elements of study design early in the paper	7-8
Setting	5	Describe the setting, locations, and relevant dates, including periods of recruitment, exposure, follow-up, and data collection	7-8
Participants	6	(*a*) Give the eligibility criteria, and the sources and methods of selection of participants	7-8
Variables	7	Clearly define all outcomes, exposures, predictors, potential confounders, and effect modifiers. Give diagnostic criteria, if applicable	7-8
Data sources/ measurement	8*	For each variable of interest, give sources of data and details of methods of assessment (measurement). Describe comparability of assessment methods if there is more than one group	*7-8*
Bias	9	Describe any efforts to address potential sources of bias	n/a
Study size	10	Explain how the study size was arrived at	n/a
Quantitative variables	11	Explain how quantitative variables were handled in the analyses. If applicable, describe which groupings were chosen and why	n/a
Statistical methods	12	(*a*) Describe all statistical methods, including those used to control for confounding	n/a
		(*b*) Describe any methods used to examine subgroups and interactions	n/a
		(*c*) Explain how missing data were addressed	n/a
		(*d*) If applicable, describe analytical methods taking account of sampling strategy	n/a
		(*e*) Describe any sensitivity analyses	n/a
**Results**			
Participants	13*	(a) Report numbers of individuals at each stage of study—eg numbers potentially eligible, examined for eligibility, confirmed eligible, included in the study, completing follow-up, and analysed	n/a
		(b) Give reasons for non-participation at each stage	n/a
		(c) Consider use of a flow diagram	
Descriptive data	14*	(a) Give characteristics of study participants (eg demographic, clinical, social) and information on exposures and potential confounders	n/a
		(b) Indicate number of participants with missing data for each variable of interest	
Outcome data	15*	Report numbers of outcome events or summary measures	n/a

For peer review only

For peer review only - http://bmjopen.bmj.com/site/about/guidelines.xhtml

Main results	16	(*a*) Give unadjusted estimates and, if applicable, confounder-adjusted estimates and their precision (eg, 95% confidence interval). Make clear which confounders were adjusted for and why they were included	n/a
		(*b*) Report category boundaries when continuous variables were categorized	n/a
		(*c*) If relevant, consider translating estimates of relative risk into absolute risk for a meaningful time period	n/a
Other analyses	17	Report other analyses done—eg analyses of subgroups and interactions, and sensitivity analyses	8-15
**Discussion**			
Key results	18	Summarise key results with reference to study objectives	16-19
Limitations	19	Discuss limitations of the study, taking into account sources of potential bias or imprecision. Discuss both direction and magnitude of any potential bias	19
Interpretation	20	Give a cautious overall interpretation of results considering objectives, limitations, multiplicity of analyses, results from similar studies, and other relevant evidence	19
Generalisability	21	Discuss the generalisability (external validity) of the study results	n/a
**Other information**			
Funding	22	Give the source of funding and the role of the funders for the present study and, if applicable, for the original study on which the present article is based	20

*Give information separately for exposed and unexposed groups.

Note: An Explanation and Elaboration article discusses each checklist item and gives methodological background and published examples of transparent reporting. The STROBE checklist is best used in conjunction with this article (freely available on the Web sites of PLoS Medicine at http://www.plosmedicine.org/, Annals of Internal Medicine at http://www.annals.org/, and Epidemiology at http://www.epidem.com/). Information on the STROBE Initiative is available at www.strobe-statement.org.

For peer review only

Practices and trends in clinical trial registration in the Pan African Clinical Trials Registry (PACTR): a descriptive analysis of registration data

Journal:	*BMJ Open*
Manuscript ID	bmjopen-2021-057474.R2
Article Type:	Original research
Date Submitted by the Author:	21-Dec-2021
Complete List of Authors:	Ndwandwe, Duduzile; South African Medical Research Council<br>Runeyi, Sinazo; South African Medical Research Council, Cochrane South Africa<br>Pienaar, Elizabeth ; South African Medical Research Council, Cochrane South Africa<br>Mathebula, Lindi; South African Medical Research Council, Cochrane SA<br>Hohlfeld, Ameer ; South African Medical Research Council, Cohrane South Africa<br>Wiysonge, Charles; South African Medical Research Council, Cochrane South Africa
**Primary Subject Heading**:	Public health
Secondary Subject Heading:	Global health, Health policy, Evidence based practice, Epidemiology, Ethics
Keywords:	Clinical trials < THERAPEUTICS, PUBLIC HEALTH, Public health < INFECTIOUS DISEASES

I, the Submitting Author has the right to grant and does grant on behalf of all authors of the Work (as defined in the below author licence), an exclusive licence and/or a non-exclusive licence for contributions from authors who are: i) UK Crown employees; ii) where BMJ has agreed a CC-BY licence shall apply, and/or iii) in accordance with the terms applicable for US Federal Government officers or employees acting as part of their official duties; on a worldwide, perpetual, irrevocable, royalty-free basis to BMJ Publishing Group Ltd ("BMJ") its licensees and where the relevant Journal is co-owned by BMJ to the co-owners of the Journal, to publish the Work in this journal and any other BMJ products and to exploit all rights, as set out in our licence.

The Submitting Author accepts and understands that any supply made under these terms is made by BMJ to the Submitting Author unless you are acting as an employee on behalf of your employer or a postgraduate student of an affiliated institution which is paying any applicable article publishing charge ("APC") for Open Access articles. Where the Submitting Author wishes to make the Work available on an Open Access basis (and intends to pay the relevant APC), the terms of reuse of such Open Access shall be governed by a Creative Commons licence – details of these licences and which Creative Commons licence will apply to this Work are set out in our licence referred to above.

Other than as permitted in any relevant BMJ Author's Self Archiving Policies, I confirm this Work has not been accepted for publication elsewhere, is not being considered for publication elsewhere and does not duplicate material already published. I confirm all authors consent to publication of this Work and authorise the granting of this licence.

For peer review only

Practices and trends in clinical trial registration in the Pan African Clinical Trials Registry (PACTR): a descriptive analysis of registration data

Duduzile Ndwandwe[1*]; Sinazo Runeyi[1]; Elizabeth D Pienaar[1]; Lindi Mathebula[1]; Ameer Hohlfeld[1]; Charles S. Wiysonge[1,2,3]

[1]Cochrane South Africa, South African Medical Research Council, Tygerberg, South Africa

[2]Division of Epidemiology and Biostatistics, Faculty of Medicine and Health Sciences, Stellenbosch University, Tygerberg, 7505, Cape Town, South Africa.

[3]School of Public Health and Family Medicine, Faculty of Health Sciences, University of Cape Town, Observatory, 7925, Cape Town, South Africa.

*Correspondence author: Duduzile Ndwandwe, Cochrane South Africa, South African Medical Research Council, Tygerberg, South Africa. Email: Duduzile.Ndwandwe@mrc.ac.za

For peer review only - http://bmjopen.bmj.com/site/about/guidelines.xhtml

Abstract

Background

The Pan African Clinical Trials Register (PACTR) is a World Health Organization (WHO) International Clinical Trials Registry Platform (ICTRP) primary register, which caters for clinical trials conducted in Africa. PACTR is the first and, at present, the only member of the Network of WHO Primary Registers in Africa. The aim is to describe and report on the trends of trial records registered in PACTR.

Methods

PACTR was established in 2007 as the AIDS, Tuberculosis, and Malaria (ATM) Clinical Trials Registry. The scope of the registry was then expanded in 2009 to include all diseases. This is a cross-sectional study of trials registered in PACTR from inception to 18 August 2021. A descriptive analysis of the use and trends of the following data fields: study intervention, disease condition, sex of the participants, sample size, ethics, funding, and availability of results conducted using Microsoft Excel.

Results

The number of trials registered has increased year on year, reaching 606 trials registered in 2020. The total number of trials registered at the time of the analysis was 2998. More than half of the trials in the registry (1655/2998 i.e. 55%) were prospectively registered. Ethical approval was received by 90% (2691/2998) of the registered trials. Factorial assignment as an intervention model was in 20% (589/2998) of the trials registered. There were 36% (1083/2998) completed trials, of which 3% (94/1083) had results available in the registry. The most dominant funding source indicated was self-funding in 23% (693/2998) of the registered trials, and 55% (1639/2998) had no funding.

For peer review only

For peer review only - http://bmjopen.bmj.com/site/about/guidelines.xhtml

Conclusion

Registration on PACTR continues to grow; however, our analysis shows that researchers' capacity-building is needed to understand the importance of the registry and how this information informs healthcare decisions. Promoting prospective trial registration remains critical to avoid selective reporting bias to inform research gaps.

Keywords: Pan African Clinical Trial Register, Clinical trial registration, Prospective trial registration

For peer review only

For peer review only - http://bmjopen.bmj.com/site/about/guidelines.xhtml

Strengths and limitations of this study

- We provided a comprehensive descriptive assessment of the trials registered in the Pan African Clinical Trial Register (PACTR)
- We conducted a descriptive analysis to assess the trends of the fields collected in the registry records to improve and prioritise activities for PACTR administration staff.
- We selected mandatory data fields to analyse to precisely assess the general trends in the trial records without analysing the free-text data captured
- There were some unavoidable missing data and variations for certain data fields, which might bias the results.

For peer review only

For peer review only - http://bmjopen.bmj.com/site/about/guidelines.xhtml

Introduction

The Pan African Clinical Trials Registry (PACTR) (www.pactr.org) was established from the AIDS, Tuberculosis, and Malaria Registry based at the South African Cochrane Centre [1-3]. The registry was established with Cochrane's Infectious Diseases Group (CIDG), based at the Liverpool School of Tropical Medicine and the World Health Organization (WHO). PACTR is the only African member of the WHO Network of Primary Registers, which transfers trial information to the WHO International Clinical Trials Registry Platform (WHO-ICTRP) (https://www.who.int/clinical-trials-registry-platform) every month [4, 5]. WHO-ICTRP serves as a platform aligned with the International Committee of Medical Journal Editors (ICMJE) for prospective trial registration. PACTR contributes to regional transparency and harmonisation of clinical trial research [6, 7] and is freely available. A database contains essential administrative and scientific information about planned, ongoing, and completed trials in a clinical trials registry [6-8]. Thus, registration of all interventional trials is considered scientific, ethical, and responsible [9-11]. Accessing clinical trials information allows informing decision-making on healthcare decisions based on all available evidence [9]. Such decisions cannot be easily made if publication bias and selective reporting exist [9].

Furthermore, the Declaration of Helsinki indicates that "Every clinical trial must be registered in a publicly accessible database before recruitment of the first subject" [9]. In the case of clinical trials, before the first participant is recruited, the information on the trial must be captured in a publicly accessible database unless the sponsor or researcher has permission to delay this to a later stage. Trial registration is one of the efforts being made to ensure transparency in clinical research, accessible patient data for subsequent analysis, and publication of results irrespective of the trial

For peer review only - http://bmjopen.bmj.com/site/about/guidelines.xhtml

outcome. This further allows for decisions related to the safety and efficacy of drugs, vaccines, and medical devices in humans, supported by the best available scientific evidence.

This, therefore, imply that clinical trial registration should advocate for prospective trial registration and that all registered trials publish their findings[12]. Trial registration further supports evidence-based medical practice, which heavily relies on available data in the public domain so that informed healthcare decisions can be made[13]. Bringing in data from clinical trials within reach of clinicians, regulators, and external stakeholders enhances the clinical trial data[13]. Prospective trial registration and subsequent results reporting are global efforts to ensure complete research transparency. Clinical trials may be registered without ethics approval, provided that recruitment of study participants has not commenced. Even Journal editors, ethics committees/institutional review boards (IRBs), regulatory authorities, and funding agencies all support the call for research transparency requiring trials to be prospectively registered[14].

There has been a push from governments and international organisations, especially since 2005, to make clinical trial information more widely available and standardise registries and registering processes. The World Health Organization (WHO) has published international Standards for Clinical Trial Registries to achieve consensus on both the minimal and the optimal operating standards for trial registration [14]. To adhere to WHO practices that ensure that collected data are not duplicated and provide meaningful information, registry staff scrutinise each application and perform regular quality checks to ensure quality data is contained in the registry.

A further benefit to registering trials prospectively in a registry is that it allows for similar or identical trials to be known, making it possible for researchers and funding agencies to avoid unnecessary duplication [7]. Also, describing clinical trials in progress makes it easier to identify research gaps for new research to advance the knowledge gaps. Registries provide quality checks

For peer review only

For peer review only - http://bmjopen.bmj.com/site/about/guidelines.xhtml

on the data submitted as part of the registration process, leading to improvements in the quality of clinical trials publicly available and pointing out potential problems early in the research design to improve clinical research conducted.

Although in the past, research on the clinical trial landscape provided key insights into the global burden of disease, and more generally, the global and regional clinical trial landscapes [4, 7, 15, 16], before PACTR, there was no regional support for longitudinal monitoring of planned and ongoing African clinical trials. PACTR is unique in recognising that African researchers face additional challenges in trial registration and seeks to provide feasible ways of overcoming these barriers [2]. PACTR has seen substantial growth in the number of trials registered from inception until recently. In this cross-sectional survey of the PACTR database, we report on the trends in the clinical trials registered.

METHODS

This was a descriptive analysis of the trends in clinical trials registered in the Pan African Clinical Trial Register (PACTR), www.pactr.org.

Data description and source

We used the WHO ICTRP, https://www.who.int/clinical-trials-registry-platform, a registry platform collating information from registries across the globe to be a one-stop portal to access clinical trial records[17]. The study used the WHO definition of a clinical trial: “any research study that prospectively assigns human participants or groups of humans to one or more health-related interventions to evaluate the effects on health outcomes”[14]. We used the advanced search function of ICTRP to identify these clinical trials registered in the PACTR on 18 August 2021.

For peer review only - http://bmjopen.bmj.com/site/about/guidelines.xhtml

Data management and analysis

Data was downloaded from WHO-ICTRP by one researcher (SR) on 18 August 2021 and exported into an excel spreadsheet. All records were quality-checked by a second researcher (DN). In each record, the following data items were used for analysis: registration status, disease condition, sex of the participants in the trials; sponsor, intervention type, funding source, the age range of participants, intervention model, phase of the trial, and overall status. We conducted a descriptive analysis of the use and trends of the registered trials in PACTR to understand the pattern of trial registration over the years using Microsoft Excel.

RESULTS

We report on the trends for trials registered in PACTR appearing in the ICTRP portal. PACTR is one of the WHO primary registers which sends data monthly to the ICTRP for one-stop to access trials records. We downloaded from the ICTRP on 18 August 2021. We used the search output to only select trials from the PACTR registry. A total of 2998 trial records were retrieved and used for analysis.

PACTR has grown substantially since its inception, with each year showing a steady increase in the number of trials registered. The year 2020 had the most registered trials (n=606). We anticipate that this increase will be seen even in 2021 (**figure 1**). **Insert Figure 1 here**

We further extrapolated the trials registered in 2020 to assess whether the significant increase was because of the COVID-19 pandemic, which has seen a rise in research activity.

Insert Figure 2 here

We found that 7% (42/606) were COVID related trials in the year 2020 and among these trials, 46% were on treatment and 20% on vaccines (figure 2).

For peer review only - http://bmjopen.bmj.com/site/about/guidelines.xhtml

153 Table 1 shows our analysis of some of the registry data items. Generally, there has been an increase in the number of trials registered in PACTR, with n= 2998 identified at the analysis time (figure1). There are 1083 (36%) completed trials, with 94 (3%) having results available in the registry. Twenty-eight percent (28%) of trials registered (836/2998) are listed as not recruiting, while 25% (755/2998) are recruiting participants. 55% (1655/2998) of the trials are registered prospectively, with the remaining 45% (1343/2998) registered retrospectively.

159 Our data show that most of the trials registered in PACTR have ethics approval (2691/2998, i.e. 90%). The intervention model refers to the general design of the strategy for assigning therapies or interventions being investigated to participants in a clinical study. Types of intervention models include single group assignment, parallel assignment, cross-over assignment, and factorial assignment. The most common intervention model in the registered trials was factorial assignment (589/2998, i.e. 20%), which means that the trial would have two (or more) intervention comparisons carried out simultaneously. The trial phases show an almost equal distribution for all clinical trial phases. We assessed the sponsor of the registered trials and reported that the sponsor could be the funder. Our data show that 55% (1639/2998) of the trials have no funding, while 23% (693/2998) are self-funded. Many trials (2240/2998, i.e. 75%) recruited both male and female participants. The median sample size was 1140.7 participants, with rannging from 0 to 1,087,000.

For peer review only

For peer review only - http://bmjopen.bmj.com/site/about/guidelines.xhtml

Table 1: Characteristics of trials in PACTR

Description	*N* (%)
**Number of trials registered**	2998
**Number of studies completed**	1083 (36.1)
**Number of completed with results**	94 (3.1)
**Overall trial status**	
Completed	1083 (36.1)
Recruiting	755 (25.2)
Not yet recruiting/pending	836 (27.9)
Recruiting	755 (25.2)
Stopped/terminated	-
Suspended	7 (0.2)
Pending	836 (27.5)
Other/unknown	317 (10.6)
**Prospective/retrospective**	
Prospectively registered	1655 (55.2)
Retrospectively registered	1343 (44.8)
**Intervention model**	
Parallel assignment	2124 (70.8)
Single group assignment	59 (2.0)
Cross-over assignment	201 (6.7)
Factorial assignment	589 (19.6)
Sequential assignment	13 (0.4)
None (open label)	12 (0.4)
**Phase**	
Not reported	2310 (77.1)
Phase I	192 (6.5)

For peer review only - http://bmjopen.bmj.com/site/about/guidelines.xhtml

Phase II	138 (4.6)
Phase III	199 (6.6)
Phase IV	155 (5.2)
**Primary Sponsor**	
University	196 (6.5)
Industry or non-governmental organisation	61 (2.0)
Government	107 (3.6)
Charities	94 (3.1)
Hospital	67 (2.2)
Self-funded	693 (23.1)
Funding agency	142 (4.7)
Other	45 (1.5)
No funding	1639 (54.7)
**Ethics approval received**	
Yes	2691 (90)
No	307 (10)
**Sex**	
Both males and females	2240 (74.7)
Female	628 (20.9)
Male	130 (4.3)
**The target number of participants**	
Minimum, maximum	0- 1, 087, 000
Mean (standard deviation)	1, 140.7 (26, 156.8)
Median (IQR)	80 (1-125)

For peer review only

For peer review only - http://bmjopen.bmj.com/site/about/guidelines.xhtml

Researchers have an option in the PACTR registry to indicate the type of intervention for the trial. We show that most common intervention type specified was drug treatment (622/2998, i.e 21%; **figure 3**).

Insert Figure 3 here

The most common disease conditions investigated in the trials conducted in PACTR registered trials were infections and infestations with 20% (586/2998), followed by the surgery category with 14% (426/2998) trials. The trials listed surgery as a disease condition included any intervention in a trial where medical and surgical care was provided. Such studies focus on diseases, injuries, and conditions affecting the abdomen, breasts, digestive system, endocrine system, and skin. Also, these trials evaluate biopsies, lab tests, and imaging tests as part of delivering care (**Figure 4**).

Insert Figure 4 here

Among the completed trials (n =1083), most of the records are without results (91%; 989/1083), and less than 10% have results (**figure 5**).

Insert Figure 5 here

The reporting section in PACTR was not mandatory until 2019. Our data show that from 2008 until 2017, results reporting was not captured. In 2018, PACTR was relaunched to include the 24-item data set required by ICTRP[14]. The reporting section is the 24th data item which collects information on the plans to share trial data and provides summary results. When PACTR was relaunched in 2018, this field was not mandatory and had options "yes," "no," and "undecided."

For peer review only

For peer review only - http://bmjopen.bmj.com/site/about/guidelines.xhtml

We, therefore, assessed whether the trials in PACTR reflected the occurrence of adding the additional data fields.

Insert Figure 6 here

The data shows that 4% (114/2998) of researchers opted to choose undecided when capturing the trial information, while 7% left this section blank (222/2998). In 2019 when the "undecided" option was removed, and the field became mandatory, there was a shift in the trend with 11% (342/2998) indicating "yes" to complete the results reporting section. This trend continues with more trials in 2020 and 2021, opting for completing the reporting section (**figure 6**).

DISCUSSION

PACTR has seen a growing number of trials registered over the years. We, therefore, report on trends in the registration of clinical trials in PACTR. Understanding the trends will allow for further improvements on the registry and identify issues that the registry team can improve on. Our data shows substantial growth in the number of registered trials over the years. PACTR registered 606 trials in 2020, contributing to 20% of the analysed trials. COVID-19 related trials only contributed to 7% of the increasing trials registered in 2020. Among these trials, 46% (19/41) being investigated, the most dominant intervention was treatment for COVID-19. This trend is on an upward trajectory as even in the year 2021, there are 15 trials related to COVID-19[18].

In our efforts to encourage prospective registration of trials, a slight shift towards prospective trial registration can be seen from 2017. Prospective trial registration is currently at 55%, while

For peer review only - http://bmjopen.bmj.com/site/about/guidelines.xhtml

retrospective trial registration is 45% (Figure 1). Trials can be registered retrospectively; however, the prospective registration of trials is encouraged to ensure transparency in research conduct, thus reducing publication and reporting bias[9]. Efforts to register trials prospectively need to be done across all primary registers. Durra et al. 2020 conducted a cross-sectional analysis of published trials registered in registries worldwide and found that prospective registration is deficient [9]. PACTR allows a trial registration if the researcher indicates when ethics approval has been applied for. We show that among the trials registered in PACTR, 90% have ethics approval which shows that the trials conducted have gone through the ethics approval process. PACTR staff also ensures that the ethics approval is verified to ensure that the data in the registry is correct.

The intervention model in PACTR registered trials indicates that registries may need to adapt to the changing trial designs, as seen with the current COVID-19 trials where adaptive trial designs were used [19-21]. Our analysis shows that the most common intervention model was factorial assignment 20%. The studies registered in PACTR show a worrying trend which shows that 55% of the trials have no funding while 23% of the trials are self-funded. Similarly, a recent cross-sectional bibliographic study showed that tuberculosis trials conducted in Africa had a dearth of financing for local African governments and NGOs [22]. There should be a shift for African governments and funders to create appropriate ways to ensure that total costs of clinical research are provided. Research institutions and universities with a real potential for success should have priority so that resources can be focused on driving research programs for Africa[23].

The other concerning trend from our analysis is that 28% of the trials are listed as not recruiting. This is indicative that researchers do not update the records, which could result in the data being misinterpreted. The “not recruiting” status indicates that participants are still receiving an intervention or being examined, but new participants are not currently recruited or enrolled.

For peer review only - http://bmjopen.bmj.com/site/about/guidelines.xhtml

However, it may also suggest that this status indicates that all participant visits are completed. The study is still open to ethics, data analysis is still ongoing, or the manuscript is pending publication. This suggests a need to build capacity on the 24-item data set [14].

Moreover, capacity building should focus on the vital role of registries as a source of data sharing, identifying research gaps, and its essential contribution in the evidence ecosystem [23] rather than another administrative activity to conduct their trials. Our data show that of the completed trials in the registry, only 3% have results available. This suggests that PACTR needs to partner with funding agencies to ensure that results are in a public domain within a specified period [24] and that clinical trial reporting is not subjected to selective reporting and publication bias[9, 25, 26].

The most common intervention in the trials conducted in Africa is treatment with drugs (21%), in which the trials registered in PACTR seek to find treatment options for infectious diseases (20%). This explains that in the most common diseases researched in Africa, there is a need for new drugs to curb the pandemics of these diseases.

The reporting section, item 24, suggests a trend towards being completed to conform to WHO-ICTRP requirements. Results reporting became mandatory in January 2019. Our analysis indicated an improvement in the reporting section completed. This trend continues with more trials in 2020 and 2021, opting for completing the reporting section. As part of our ongoing analysis, this analysis shows that more needs to be done to build capacity on clinical trials through partnering with regulatory, sponsors, and researchers to ensure that clinical research conducted in Africa meets the global standards.

Limitations

For peer review only - http://bmjopen.bmj.com/site/about/guidelines.xhtml

We conducted a descriptive analysis of the data to analyse what is happening in the registry. We used the ICTRP platform to search for PACTR registered studies. This approach may have excluded trials currently being processed at the time of downloading this data file which may be prior to sending montly data file to ICTRP, thus underrepresenting the total number of registered trials. The disease category was too vast to understand the specific disease conditions investigated when analyzing the data. In the future, we will focus on unpacking the disease categories and understanding the trends in the diseases being evaluated. Also, there are data elements in which a researcher would indicate "other," resulting in many trials with "other" as a disease condition. Assessment of the data allows the PACTR review team to reinforce correct data entry when conducting reviews of the submitted trials and include all mandatory data fields required by WHO. Our analysis did not have the free text data captured in the registry, which will need further unpacking to understand the trends of trial registration. The description of a sponsor into categories may limit what the researcher identifies as a sponsor to how we classified the sponsors leading to some variation.

Conclusion

Registration on PACTR has continued to grow since 2008. PACTR provides valuable data to map clinical trial conduct on the African continent. More work needs to be done to ensure that, as the registry team, we guarantee capacity building in collaboration with the ethics committee, funders, and sponsors to provide that PACTR ensures that clinical research conducted in Africa meets international standards. There is an urgent need to continue to raise awareness for prospective trial registration and reporting of summary results. This will allow researchers to understand the importance of data sharing to contribute to research gaps to find solutions for Africa.

For peer review only - http://bmjopen.bmj.com/site/about/guidelines.xhtml

Further considerations

PACTR should expand its efforts to build capacity in the African continent, explicitly creating links with ethics committees to evaluate the underlying quality of the scientific data included in these trials. We noted several instances where ethics documents submitted by the researcher needed to be verified to confirm that the ethics approval received is legit. Furthermore, research is required to understand the reasons for enforcing trial registration requirements by editors, funders, and regulatory bodies across the continent.

Patient and public involvement

No patient or public involvement.

Funding

The publication cost for this article is supported with funds from the South African Medical Research Council (project code 43500).

Acknowledgments

The authors acknowledges the South African Medical Research Council for funding support. The views expressed in this article are those of the authors. They do not necessarily reflect the opinions or policies of the South African Medical Research Council, Cochrane, or other institutions that the authors are affiliated with.

Ethics approval We conducted an analysis of publicly available clinical trials information. Therefore ethics approval was not required.

For peer review only

For peer review only - http://bmjopen.bmj.com/site/about/guidelines.xhtml

Competing interests

The authors declare that they have no competing interests.

Author Contributions

DN wrote the first draft, coordinated and integrated comments from co-authors, approved the final version for publication, and is the guarantor of the manuscript. SR and LM conducted the analysis of the data. SR, LM, EP, AH, CSW critically revised successive drafts of the manuscript, provided important intellectual input, and approved the final version of the manuscript. The authors have read and approved the article's final version for submission.

Data availability statement.

The data underlying the study will be made available to other researchers upon request. A file of the data will be shared. Additionally, the publication will be shared on the PACTR registry database and shared with the WHO ICTRP.

List of figures

Figure 1: Number of retrospective and prospective registrations on PACTR by year. We conducted a descriptive analysis of the trials registered in PACTR and showed the number of trials registered per year on the x axis. The orange bar represents trials flagged as prospective registration, and the blue bar represents trials flagged as retrospective upon registration. The y axis represents the number of trials.

For peer review only

For peer review only - http://bmjopen.bmj.com/site/about/guidelines.xhtml

Figure 2: An assessment of the trials registered in the year 2020. We describe the number of trials registered in 2020 presented as a pie chart indicated in orange and green colours. The green pie represents the COVID-19 trials which are further expanded to show the different interventions of these trials in different colour shades.

Figure 3: Type of intervention used for the registered trials in PACTR. The trials registered indicate the intervention being investigated in their record. We describe the intervention of all the trials registered at the time of analysis. The results are represented as a bar graph indicating the number of trials with a specific intervention. The total number of trials for a particular intervention is presented at the top of each bar.

Figure 4: Disease conditions investigated in PACTR registered trials. An investigation of the disease categories is represented as bar graphs on the y axis. The number of trials registered to investigate a specific disease condition is presented on the bar.

Figure 5: Completed trials with results in PACTR. We describe the number of registered trials with a "complete" status represented with the red bar and the actual number of the trials presented as N at the bottom of the bar. The blue bar represents the number of trials with available results, and the grey bar represents the completed trials without results.

Figure 6: Trends in results reporting over the years. We describe the reporting section of the registered trials from 2008 to 2021 presented in bars. The dark green bar indicates trial records in

For peer review only - http://bmjopen.bmj.com/site/about/guidelines.xhtml

which the reporting section was not completed. The lighter green represents the trials that indicated "no" to reporting results. Light blue indicates registered trials with results reported. The yellow bar represents the records of undecided results to report the results. The y axis is the total number of trials.

REFERENCES:

1. Abrams A, Siegfried N: **The Pan African Clinical Trials Registry: year one data analysis of the only African member of the World Health Organization Network of Primary Registries**. *J Evid Based Med* 2010, **3**(4):195-200.
2. Abrams A, Siegfried N: **Growing everyday: the Pan African Clinical Trials Registry**. *J Evid Based Med* 2011, **4**(3):172-178.
3. Abrams AL: **One of a kind--the Pan African Clinical Trials Registry, a regional registry for Africa**. *Pan Afr Med J* 2011, **9**:42.
4. Isaakidis P, Swingler GH, Pienaar E, Volmink J, Ioannidis JP: **Relation between burden of disease and randomised evidence in sub-Saharan Africa: survey of research**. *BMJ* 2002, **324**(7339):702.
5. Abrams A, Siegfried N: **A Pan African Clinical Trials Registry for the specific needs of triallists on the continent**. *S Afr Med J* 2010, **100**(5):294-295.
6. Sim I, Chan AW, Gulmezoglu AM, Evans T, Pang T: **Clinical trial registration: transparency is the watchword**. *Lancet* 2006, **367**(9523):1631-1633.
7. Swingler GH, Pillay V, Pienaar ED, Ioannidis JP: **International collaboration, funding and association with burden of disease in randomized controlled trials in Africa**. *Bull World Health Organ* 2005, **83**(7):511-517.
8. Gulmezoglu AM, Sim I: **International clinical trial registration: any progress?** *Wien Klin Wochenschr* 2006, **118**(1-2):1-2.
9. Al-Durra M, Nolan RP, Seto E, Cafazzo JA: **Prospective registration and reporting of trial number in randomised clinical trials: global cross sectional study of the adoption of ICMJE and Declaration of Helsinki recommendations**. *BMJ* 2020, **369**:m982.
10. De Angelis C, Drazen JM, Frizelle FA, Haug C, Hoey J, Horton R, Kotzin S, Laine C, Marusic A, Overbeke AJ *et al*: **[Clinical trial registration: a statement from the International Committee of Medical Journal Editors]**. *Tidsskrift for den Norske laegeforening : tidsskrift for praktisk medicin, ny raekke* 2004, **124**(18):2339.
11. Zarin DA, Tse T, Williams RJ, Rajakannan T: **Update on Trial Registration 11 Years after the ICMJE Policy Was Established**. *The New England journal of medicine* 2017, **376**(4):383-391.

For peer review only

For peer review only - http://bmjopen.bmj.com/site/about/guidelines.xhtml

12. Authority HR: **Research Transparency** In. Edited by Authority NR. 2 Redman Place, Stratford, London, E20 1JQ; 2020.
13. Joshi M, Bhardwaj P: **Impact of data transparency: Scientific publications**. *Perspectives in clinical research* 2018, **9**(1):31-36.
14. **International Standards for Clinical Trial Registries** [https://apps.who.int/iris/bitstream/handle/10665/274994/9789241514743-eng.pdf]
15. Isaakidis P, Ioannidis JP: **Evaluation of cluster randomized controlled trials in sub-Saharan Africa**. *Am J Epidemiol* 2003, **158**(9):921-926.
16. Swingler GH, Volmink J, Ioannidis JP: **Number of published systematic reviews and global burden of disease: database analysis**. *BMJ* 2003, **327**(7423):1083-1084.
17. Ghersi D, Pang T: **En route to international clinical trial transparency**. *Lancet* 2008, **372**(9649):1531-1532.
18. Tsanni A: **African scientists race to test COVID drugs - but face major hurdles**. In: *Nature* vol. 599; 2021: 25-27
19. Wang SJ: **Recent advances in clinical trial design considerations in Thera"nostics"**. *Contemporary clinical trials* 2020, **96**:106100.
20. Lienhardt C, Nunn A, Chaisson R, Vernon AA, Zignol M, Nahid P, Delaporte E, Kasaeva T: **Advances in clinical trial design: Weaving tomorrow's TB treatments**. *PLoS medicine* 2020, **17**(2):e1003059.
21. Raber-Johnson ML, Gallwitz WE, Sullivan EJ, Storer P: **Innovation in Clinical Trial Design and Product Promotion: Evolving the Patient Perspective With Regulatory and Technological Advances**. *Therapeutic innovation & regulatory science* 2020, **54**(3):519-527.
22. Hohlfeld ASJ, Mathebula L, Pienaar ED, Abrams A, Lutje V, Ndwandwe D, Kredo T: **Tuberculosis treatment intervention trials in Africa: A cross-sectional bibliographic study and spatial analysis**. *PloS one* 2021, **16**(3):e0248621.
23. Whitworth JA, Kokwaro G, Kinyanjui S, Snewin VA, Tanner M, Walport M, Sewankambo N: **Strengthening capacity for health research in Africa**. *Lancet* 2008, **372**(9649):1590-1593.
24. Knowles RL, Ha KP, Mueller J, Rawle F, Parker R: **Challenges for funders in monitoring compliance with policies on clinical trials registration and reporting: analysis of funding and registry data in the UK**. *BMJ open* 2020, **10**(2):e035283.
25. Edem B, Onwuchekwa C, Wariri O, Nkereuwem E, Nkereuwem OO, Williams V: **Trends in clinical trial registration in sub-Saharan Africa between 2010 and 2020: a cross-sectional review of three clinical trial registries**. *Trials* 2021, **22**(1):472.
26. Hart B, Lundh A, Bero L: **Effect of reporting bias on meta-analyses of drug trials: reanalysis of meta-analyses**. *BMJ* 2012, **344**:d7202.

For peer review only

For peer review only - http://bmjopen.bmj.com/site/about/guidelines.xhtml

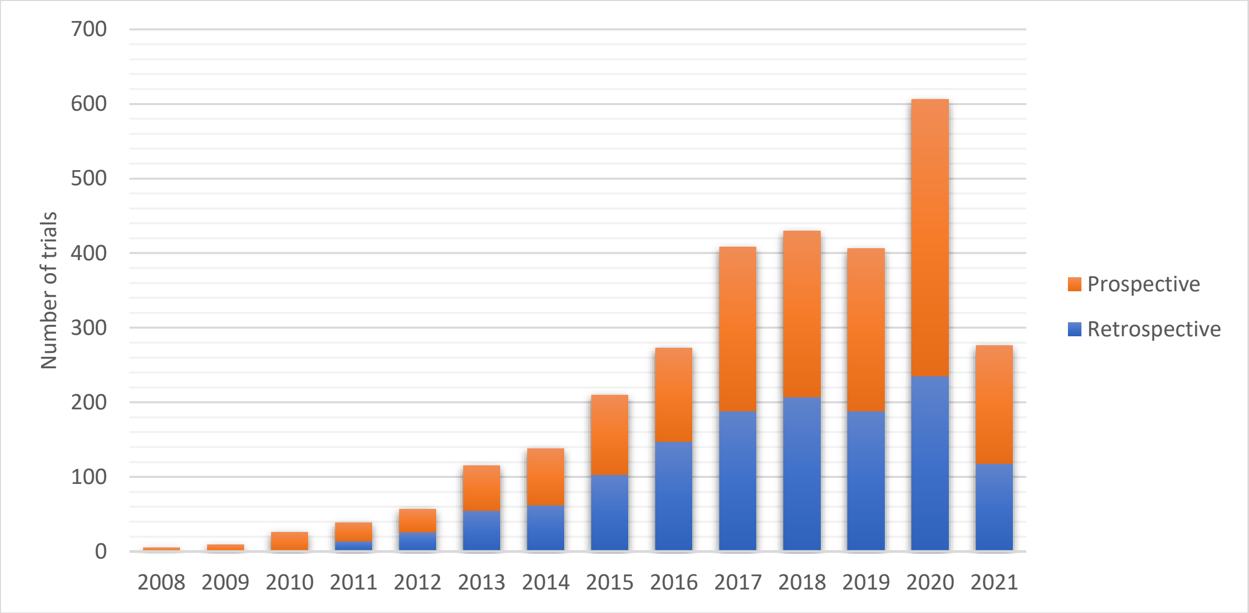

Figure 1: Number of retrospective and prospective registrations on PACTR by year

For peer review only

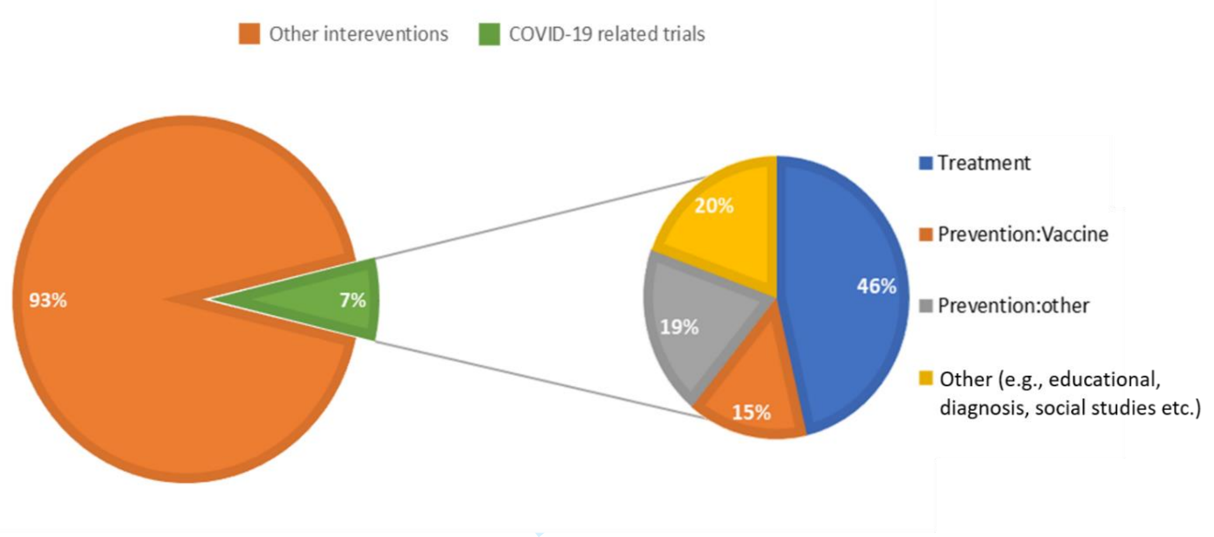

Figure 2: An assessment of the trials registered in the year 2020.

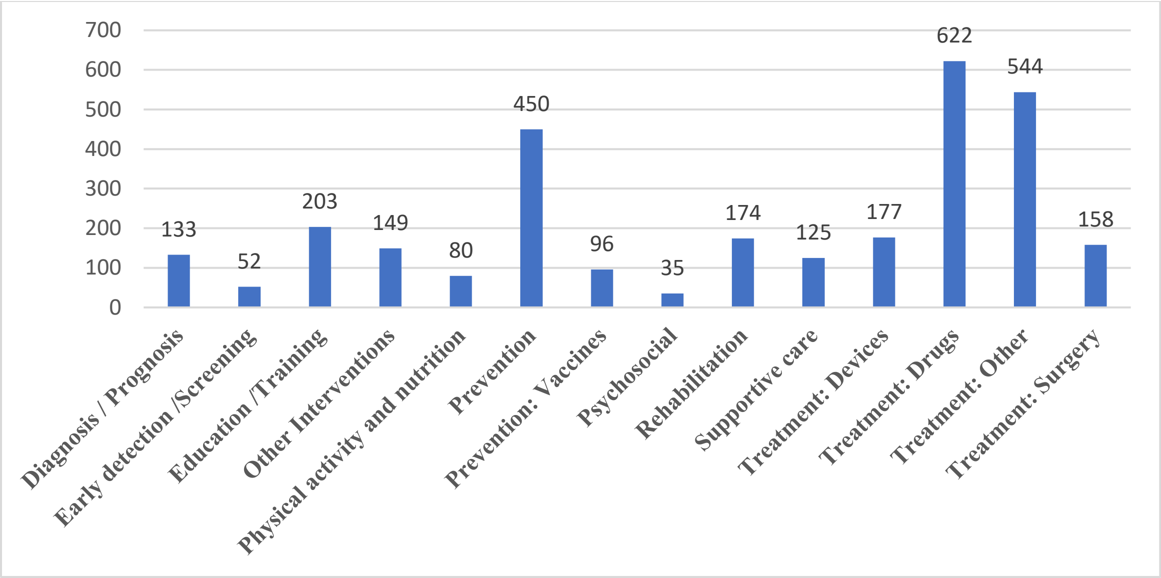

Figure 3: Type of intervention used for the registered trials in PACTR

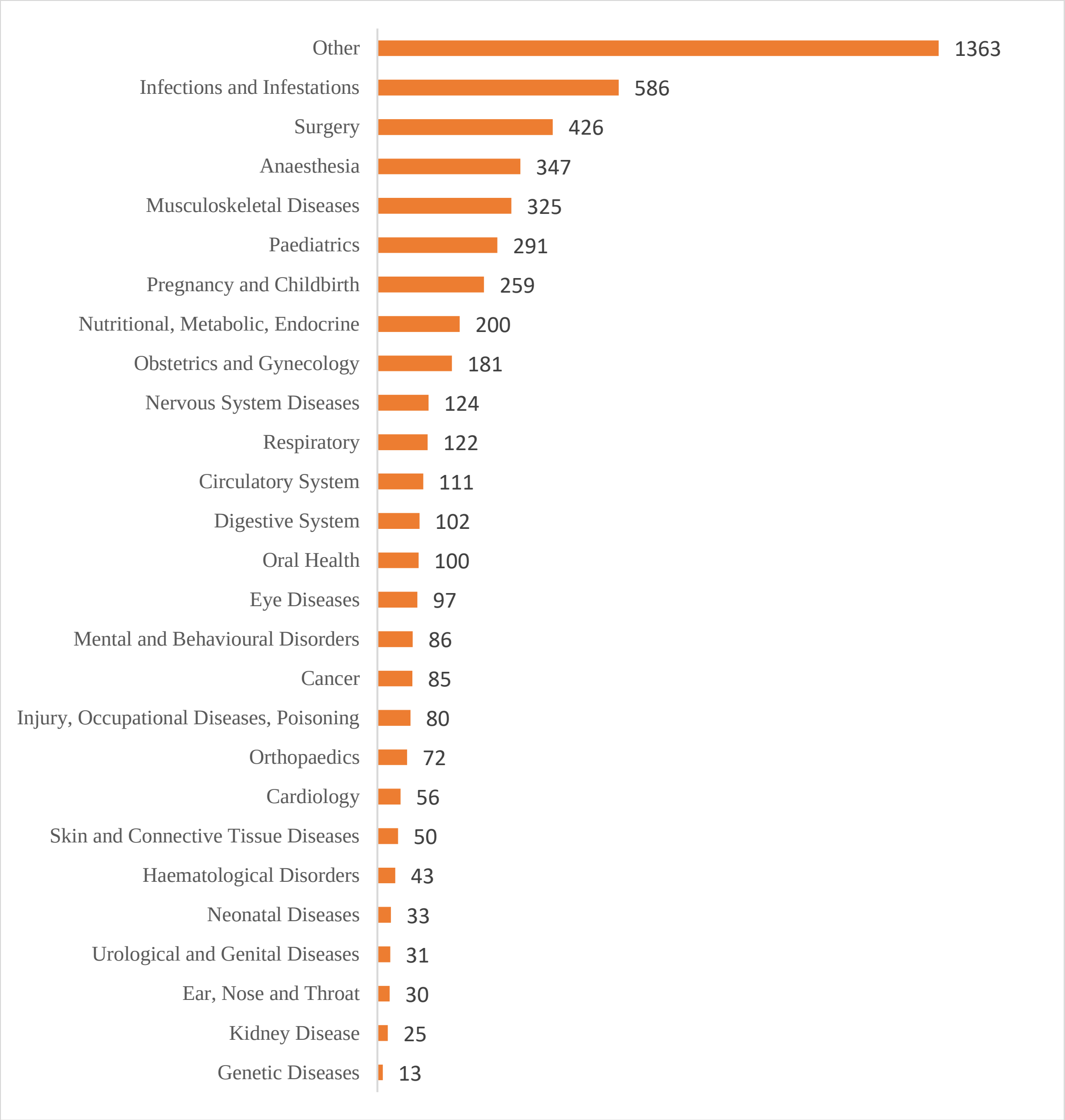

Figure 4: Disease conditions investigated in PACTR registered trials

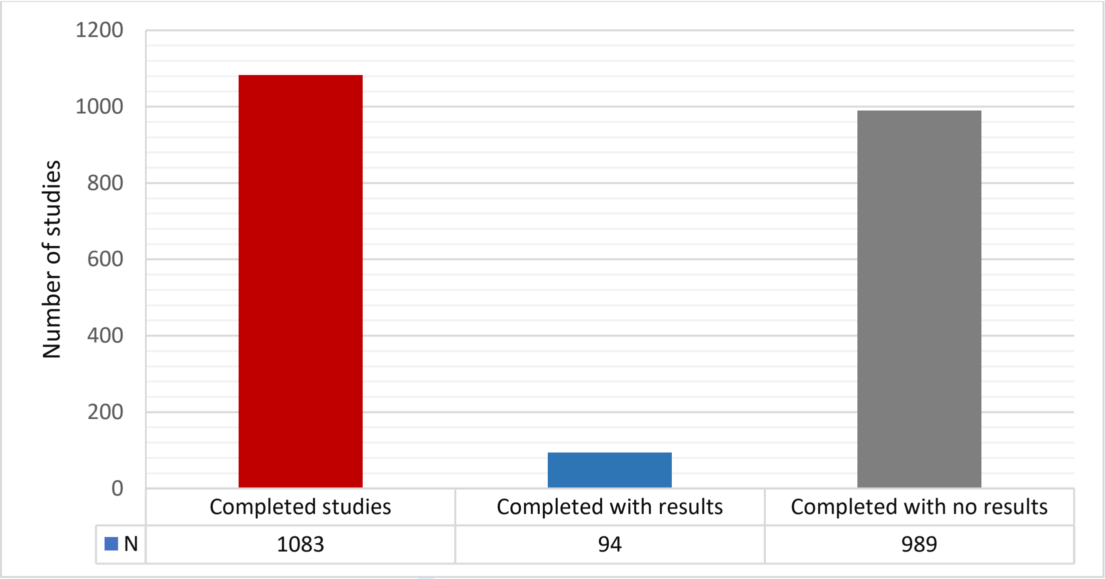

Figure 5: Completed trials with results in PACTR

peer review only

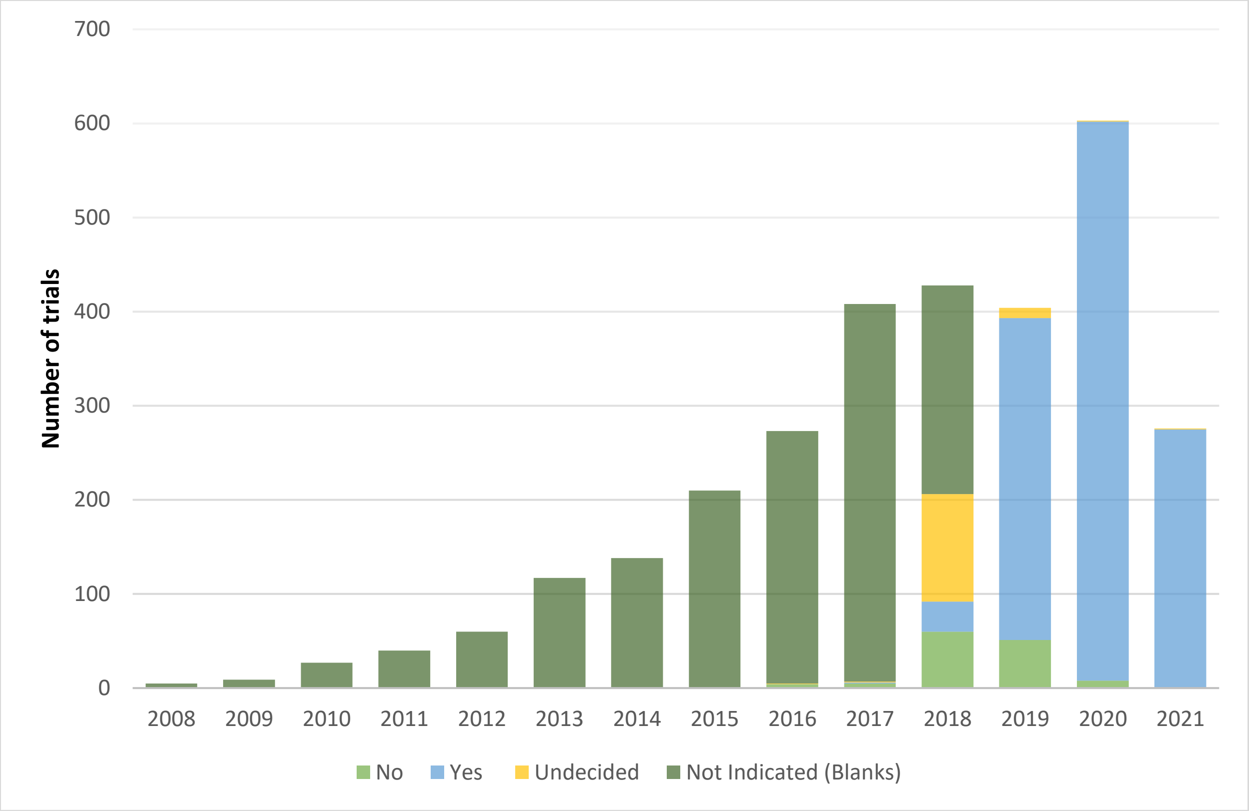

Figure 6: Trends in results reporting over the years

Rotavirus vaccine clinical trials: a cross sectional analysis of clinical trials registries

STROBE Statement—Checklist of items that should be included in reports of ***cross-sectional studies***

	Item No	Recommendation	Page No
**Title and abstract**	1	(*a*) Indicate the study's design with a commonly used term in the title or the abstract	1
		(*b*) Provide in the abstract an informative and balanced summary of what was done and what was found	2-3
**Introduction**			
Background/rationale	2	Explain the scientific background and rationale for the investigation being reported	4-6
Objectives	3	State specific objectives, including any prespecified hypotheses	6
**Methods**			
Study design	4	Present key elements of study design early in the paper	7-8
Setting	5	Describe the setting, locations, and relevant dates, including periods of recruitment, exposure, follow-up, and data collection	7-8
Participants	6	(*a*) Give the eligibility criteria, and the sources and methods of selection of participants	7-8
Variables	7	Clearly define all outcomes, exposures, predictors, potential confounders, and effect modifiers. Give diagnostic criteria, if applicable	7-8
Data sources/ measurement	8*	For each variable of interest, give sources of data and details of methods of assessment (measurement). Describe comparability of assessment methods if there is more than one group	*7-8*
Bias	9	Describe any efforts to address potential sources of bias	n/a
Study size	10	Explain how the study size was arrived at	n/a
Quantitative variables	11	Explain how quantitative variables were handled in the analyses. If applicable, describe which groupings were chosen and why	n/a
Statistical methods	12	(*a*) Describe all statistical methods, including those used to control for confounding	n/a
		(*b*) Describe any methods used to examine subgroups and interactions	n/a
		(*c*) Explain how missing data were addressed	n/a
		(*d*) If applicable, describe analytical methods taking account of sampling strategy	n/a
		(*e*) Describe any sensitivity analyses	n/a
**Results**			
Participants	13*	(a) Report numbers of individuals at each stage of study—eg numbers potentially eligible, examined for eligibility, confirmed eligible, included in the study, completing follow-up, and analysed	n/a
		(b) Give reasons for non-participation at each stage	n/a
		(c) Consider use of a flow diagram	
Descriptive data	14*	(a) Give characteristics of study participants (eg demographic, clinical, social) and information on exposures and potential confounders	n/a
		(b) Indicate number of participants with missing data for each variable of interest	
Outcome data	15*	Report numbers of outcome events or summary measures	n/a

For peer review only - http://bmjopen.bmj.com/site/about/guidelines.xhtml

Main results	16	(*a*) Give unadjusted estimates and, if applicable, confounder-adjusted estimates and their precision (eg, 95% confidence interval). Make clear which confounders were adjusted for and why they were included	n/a
		(*b*) Report category boundaries when continuous variables were categorized	n/a
		(*c*) If relevant, consider translating estimates of relative risk into absolute risk for a meaningful time period	n/a
Other analyses	17	Report other analyses done—eg analyses of subgroups and interactions, and sensitivity analyses	8-15
**Discussion**			
Key results	18	Summarise key results with reference to study objectives	16-19
Limitations	19	Discuss limitations of the study, taking into account sources of potential bias or imprecision. Discuss both direction and magnitude of any potential bias	19
Interpretation	20	Give a cautious overall interpretation of results considering objectives, limitations, multiplicity of analyses, results from similar studies, and other relevant evidence	19
Generalisability	21	Discuss the generalisability (external validity) of the study results	n/a
**Other information**			
Funding	22	Give the source of funding and the role of the funders for the present study and, if applicable, for the original study on which the present article is based	20

*Give information separately for exposed and unexposed groups.

Note: An Explanation and Elaboration article discusses each checklist item and gives methodological background and published examples of transparent reporting. The STROBE checklist is best used in conjunction with this article (freely available on the Web sites of PLoS Medicine at http://www.plosmedicine.org/, Annals of Internal Medicine at http://www.annals.org/, and Epidemiology at http://www.epidem.com/). Information on the STROBE Initiative is available at www.strobe-statement.org.

For peer review only
